# CUL5-ARIH2 E3-E3 ubiquitin ligase structure reveals cullin-specific NEDD8 activation

**DOI:** 10.1038/s41589-021-00858-8

**Published:** 2021-09-13

**Authors:** Sebastian Kostrhon, J. Rajan Prabu, Kheewoong Baek, Daniel Horn-Ghetko, Susanne von Gronau, Maren Klügel, Jérôme Basquin, Arno F. Alpi, Brenda A. Schulman

**Affiliations:** 1 Department of Molecular Machines and Signaling, Max Planck Institute of Biochemistry, Martinsried, Germany; 2 Department of Structural Cell Biology, Max Planck Institute of Biochemistry, Martinsried, Germany

**Keywords:** Enzymes, Post-translational modifications

## Abstract

An emerging mechanism of ubiquitylation involves partnering of two distinct E3 ligases. In the best-characterized E3-E3 pathways, ARIH-family RING-between-RING (RBR) E3s ligate ubiquitin to substrates of neddylated cullin-RING E3s. The E3 ARIH2 has been implicated in ubiquitylation of substrates of neddylated CUL5-RBX2-based E3s, including APOBEC3-family substrates of the host E3 hijacked by HIV-1 virion infectivity factor (Vif). However, the structural mechanisms remained elusive. Here structural and biochemical analyses reveal distinctive ARIH2 autoinhibition, and activation on assembly with neddylated CUL5-RBX2. Comparison to structures of E3-E3 assemblies comprising ARIH1 and neddylated CUL1-RBX1-based E3s shows cullin-specific regulation by NEDD8. Whereas CUL1-linked NEDD8 directly recruits ARIH1, CUL5-linked NEDD8 does not bind ARIH2. Instead, the data reveal an allosteric mechanism. NEDD8 uniquely contacts covalently linked CUL5, and elicits structural rearrangements that unveil cryptic ARIH2-binding sites. The data reveal how a ubiquitin-like protein induces protein-protein interactions indirectly, through allostery. Allosteric specificity of ubiquitin-like protein modifications may offer opportunities for therapeutic targeting.

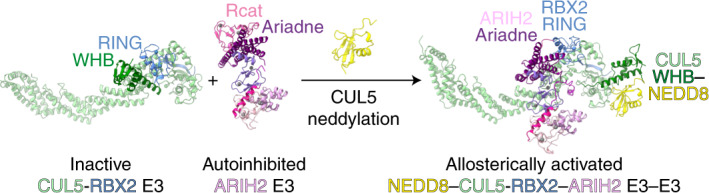

## Abstract (web summary)

Structures and biochemistry reveal how covalent linkage of a ubiquitin-like protein elicits protein–protein interactions indirectly. NEDD8 allosterically activates CUL5-RBX2 cullin-RING E3 binding to the ARIH2 RBR-type E3 for joint ubiquitylation.

## Main

Ubiquitin (UB) and ubiquitin-like proteins (UBLs) are eukaryotic posttranslational modifiers that determine the functions and fates of many proteins. Key facets of this regulation are: (1) E3 ligase-mediated linkage of the C terminus of UB or UBL to a target protein; and (2) recognition of the modified protein by a specific UB- or UBL-binding partner^1,2^.

The UBL NEDD8 is nearly 60% identical to UB, but has distinct targets and functions^3^. The best-characterized regulation by NEDD8 involves its linkage to a conserved lysine in cullin proteins^4^. Cullins (CULs) partner with RING-box-protein (RBX) RING-type proteins to form core scaffolds within multiprotein cullin-RING E3 UB ligases (CRLs). In mammalian cells, CUL1, CUL2, CUL3 and CUL4 form dedicated core complexes with RBX1, while CUL5 partners with RBX2 (refs. ^5–11^). Cullin and RBX proteins interact via an intermolecular β-sheet involving the cullin α/β-domain and RBX N-terminal region^5^. These elements are thought to fold on binding to each other^5^. We refer to the intermolecular domain as ‘C/R’ due to its containing elements from both the cullin and the RBX protein. C/R domains are sufficiently homologous across the cullin and RBX families to allow recombinant generation of alternative combinations, for example CUL5-RBX1 and CUL1-RBX2. Such alternative CUL-RBX pairings have proven useful for mechanistic studies^7,12^, although it is unclear if they normally exist and function in vivo.

CRLs assemble and perform ubiquitylation when opposite ends of a CUL-RBX core scaffold associate with interchangeable substrate-bound receptors and catalytic UB-carrying enzymes^4,13^. Suites of substrate-binding receptors—often in complex with adapter proteins—associate with cognate cullin N-terminal domains^4^. For example, around 70 human F-box proteins have an F-box motif, which binds the adapter protein SKP1, which in turn binds CUL1’s N-terminal domain. Most F-box proteins also have distinct protein–protein interaction domains that recruit substrates to the CUL1-RBX1 core. Meanwhile, around 40 substrate-binding BC-box proteins bind the Elongin B–Elongin C (ELOBC) complex, which is an adapter for CUL5-RBX2. Individual CRLs are named based on cullin identity, with substrate receptor denoted in superscript. For example, CRL5^ASB9^ refers to a CUL5-RBX2 complex with the ELOBC adapter and the BC-box substrate receptor ASB9 (refs. ^14,15^).

Opposite to the substrate receptor-binding end of a CRL, the C-terminal domains of the cullin and RBX proteins mediate ubiquitylation. The cullin’s C terminus consists of a rod-like H29-helix that continues into the winged-helix B (WHB) domain containing the neddylation site^3,5^. The C terminus of the RBX protein is the hallmark E3 ligase RING domain, which in the context of a neddylated CRL can bind various UB-carrying enzymes—E2s in the UBE2D, UBE2G and UBE2R families, and ARIH-family RBR E3s—from which UB is transferred to a receptor-bound substrate^9,13,16,17^. Structures representing RING E3-E2~UB conjugates (‘~’ refers to thioester bond between UB’s C terminus and an enzyme catalytic cysteine) were defined nearly a decade ago^18–20^. Moreover, a recent cryogenic-electron microscopy (cryo-EM) structure showed how the RBX1 RING-bound UBE2D~UB active site is juxtaposed with substrates of neddylated CRL1^β-TRCP17^. However, mechanisms underlying assembly between neddylated CRL E3s and ARIH-family RBR E3s are only beginning to emerge^9,16,21–23^.

ARIH-family E3s—like many RBR ligases—are autoinhibited on their own^24–28^. ARIH-family E3s are allosterically activated on assembly with a neddylated CRL into an E3-E3 ligase^9,16,21,23^. These E3-E3 ligases promote a UB transfer cascade: UB is transferred from the E2 enzyme UBE2L3 to the catalytic cysteine of the neddylated CRL E3-bound ARIH E3, and then from the ARIH E3 to the CRL E3-bound substrate^9,16^.

E3-E3 ligase formation requires cullin neddylation, and is remarkably specific: RBX1-containing neddylated CRLs partner with ARIH1, whereas neddylated CRL5s partner with ARIH2 (refs. ^9,16,21,23^). Cryo-EM structures have shown how neddylated CRL1s use ARIH1 to ubiquitylate F-box protein-bound substrates^23^. However, several distinctive features suggested unique NEDD8 regulation of the CRL5 assembly with ARIH2. In cells, CUL5 is not neddylated by the enzymes that typically modify CULs 1–4 (ref. ^7^). Instead, CUL5 neddylation requires RBX2 and the metazoan-specific NEDD8 E2 UBE2F^7^. The importance of CUL5-RBX2-specific regulation is underscored by its pathological hijacking by HIV-1. HIV-1 replication depends on redirecting cellular ubiquitylation pathways to degrade host restriction factors^29^. HIV-1 Vif conscripts the host protein CBFβ to form a heterodimeric BC-box receptor, which assembles into a CRL5^Vif-CBFβ^ E3 that ubiquitylates APOBEC3-family restriction factors^30–32^. Vif-mediated APOPEC3 degradation—and HIV-1 infectivity—require neddylation, UBE2F, CUL5, RBX2 and ARIH2 (refs. ^8,9^). Similarly, the human CRL5^ASB9^ E3 uses ARIH2 and not ARIH1 to ubiquitylate its substrate creatine kinase B (CKB)^9,22,33^. From a structural perspective, the interactions between NEDD8 and CUL1’s WHB domain observed in recent cryo-EM structures differ from those between NEDD8 and a CUL5 fragment in a previous crystal structure^12,17,23^. Moreover, hydrogen–deuterium exchange (HDX) data for a neddylated CRL5-ARIH2 complex are incompatible with the structurally characterized assemblies between neddylated CRL1s and ARIH1 (refs. ^23,33^). Thus, we performed structural and biochemical studies to gain insights into the distinctive assembly between ARIH2 and neddylated CRL5s.

## Results

### Crystal structure of autoinhibited ARIH2

**Fig. 1:**
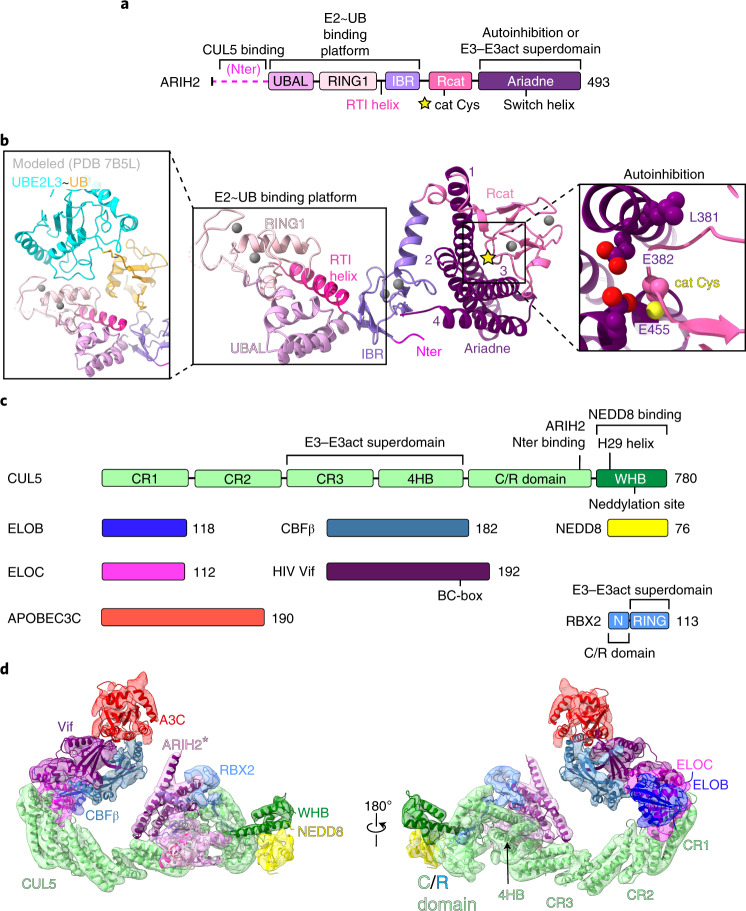
**a**, ARIH2 schematic color-coded by domains. The catalytic Cys310 (cat Cys) in the Rcat domain is indicated with a yellow star. Nter, N terminus. **b**, Crystal structure of autoinhibited ARIH2 (residues 51–493) is shown in center, with domains colored as in **a**, zinc atoms as spheres and Ariadne domain helices numbered. The ARIH2 UBAL, RING1, RTI helix and IBR domains form an E2~UB-binding platform. Left inset, UBE2L3~UB (from complex with neddylated CRL1-bound ARIH1, ref. ^23^) modeled onto the ARIH2 E2~UB-binding platform. Right inset, close-up (rotated 30° in *x* and 30° in *y*) highlighting L381, E382 and E455 mediating autoinhibition. ARIH2 catalytic Cys thiol is shown as a yellow sphere. **c**, Color-coded schematics of subunits and domains of neddylated CRL5^Vif-CBFβ^ and APOBEC3C (A3C). **d**, Model of ARIH2* (full-length ARIH2 with L381A, E382A and E455A residue substitutions) complex with neddylated CRL5^Vif-CBFβ^ and A3C in cryo-EM reconstruction low-pass filtered to 7.5 Å. Coordinates for ARIH2*, RBX2 and a portion of neddylated CUL5 (CR3 domain through the C terminus) were built into the map shown in Extended Data Fig. 2b. Structures of A3C^39^ and Vif-CBFβ-ELOBC-CUL5 N-terminal domain^36^ were fit in density.

To understand how ARIH2 is regulated, we determined the 2.45 Å resolution crystal structure of a near full-length, autoinhibited version that lacks the N-terminal region predicted to be disordered^21^ (Fig. 1a,b and Supplementary Table 1). The two ARIH2 molecules in the asymmetric unit superimpose with 0.6 Å root mean square deviation (r.m.s.d.), hence only one is described. The canonical RBR E3 catalytic elements (RING1, RTI helix, in-between RING (IBR) and Rcat domains) are interspersed with the ARIH-specific UBA-like (UBAL) and Ariadne domains in a two-part arrangement. One part is a platform containing the canonical RBR E2~UB-binding surfaces (Fig. 1b). Studies representing other, active RBR E3s revealed that RING1 binds the E2, while UB is cradled in an adjacent bowl-shaped surface formed by RING1, RTI helix and IBR domains^23,34,35^. To show roles of these elements from ARIH2, a model was generated by overlaying the structure of an ARIH1-UBE2L3~UB complex (Fig. 1b)^23^. The arrangement of the RING1, RTI helix and IBR domains in autoinhibited ARIH2 resembles that in activated ARIH1 bound to UBE2L3~UB. ARIH2’s UBAL domain stabilizes the E2~UB-binding platform on the opposite side, by intercalating between the RING1 and IBR domains, and packing against the RTI helix.

**Extended Data Fig. 1:**
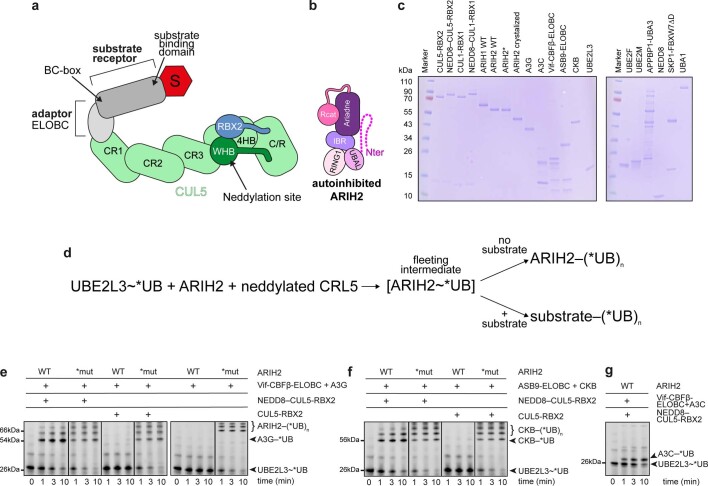
ARIH2 and neddylated CRL5 E3-E3 assembly and activity. **a**, Cartoon representing the core CRL5 assembly, highlighting relative locations of domains. **b**, Cartoon representing structure of autoinhibited ARIH2 showing relative locations of domains. **c**, Coomassie-stained SDS-PAGE gel of proteins used in this study. **d**, Schematic of chase portion of pulse-chase assays of E3-E3 activity. The assays detect fluorescent UB (*UB) harboring an N-terminal tag wherein a cysteine is conjugated to fluorescein-5-maleimide. First, an E1 (UBA1)-catalyzed pulse reaction generates the reactive, thioester-linked UBE2L3~*UB intermediate. After the pulse reaction is quenched, the chase is initiated by adding ARIH2 and neddylated CRL5, which mediate *UB transfer from UBE2L3. ARIH2 is activated by binding a neddylated CRL5. When a CRL5 substrate is added, E3-E3 activity is monitored by transfer of *UB from UBE2L3 to the substrate via a fleeting thioester-linked ARIH2~*UB intermediate that is not readily detected. Without a CRL5 substrate, intrinsic E3-E3 activity is detected by ARIH2 autoubiquitylation. ~ refers to thioester linkage. – refers to isopeptide linkage. **e**, Fluorescent scans of gels examining ARIH2 and CRL5^Vif-CBFβ^-dependent *UB transfer from UBE2L3 through E3-E3 cascade for ubiquitylating A3G substrate, following assay scheme shown in d. The assays test effects of NEDD8 and/or mutationally overcoming ARIH2 autoinhibition. The indicated E3-E3 components were added to UBE2L3~*UB. Components of CRL5^Vif-CBFβ^ were added as two subcomplexes: either neddylated or unneddylated CUL5-RBX2, and Vif–CBFβ-ELOBC mixed with A3G substrate. Reactions use either WT ARIH2 or ARIH2* (*mut, with L381A E382A E455A mutations). Results are representative of N = 2 independent experiments. Assay results shown in the main figures are quantified from the 10-minute time point and normalized relative to activity with WT proteins (Source Data Extended Data Fig. 1). **f**, Experiment performed as in e, except with CRL5^ASB9^ and substrate CKB. **g**, Experiment performed as in e, except with substrate A3C. Source data

The second part of the ARIH2 structure shows autoinhibition: the Ariadne domain binds the active site in the catalytic Rcat domain (Fig. 1b). The Ariadne domain is an elongated four-helix bundle. A groove between the first and third helices of the Ariadne domain secures the catalytic cysteine loop from the Rcat domain. In particular, Ariadne domain residues Leu381, Glu382 and Glu455, respectively, contact the beginning, middle and end of the Rcat domain catalytic cysteine loop. A triple Leu381Ala, Glu382Ala, Glu455Ala mutant, which we term ARIH2*, was relieved of autoinhibition as monitored by autoubiquitylation. ARIH2* maintained ability to ubiquitylate substrates of CRL5^ASB9^ and CRL5^Vif-CBFβ^ (Extended Data Fig. 1). ARIH2*-mediated ubiquitylation of a CRL5 substrate required CUL5-RBX2 (Extended Data Fig. 1e,f).

### Overall E3-E3 assembly between ARIH2 and neddylated CRL5s

**Extended Data Fig. 2:**
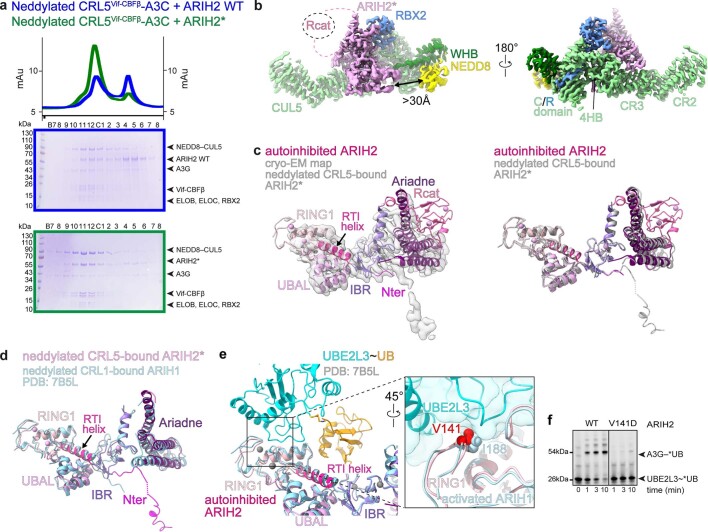
Interactions of ARIH2 and ARIH2*. **a**, Chromatograms (top) and Coomassie-stained SDS-PAGE gels of fractions (bottom) from size exclusion chromatography of neddylated CRL5^Vif-CBFβ^–A3C mixed with WT ARIH2 (blue) or ARIH2* (green, Source Data Extended Data Fig. 2). The experiment was performed twice. **b**, Interactions between ARIH2* (violet) and neddylated CUL5-RBX2 seen in map from ARIH2*-neddylated CRL5^Vif-CBFβ^-A3C complex generated using DeepEMhancer^40^. Connections to the ARIH2* Rcat domain, not visible in the map, are indicated as dotted lines. Black arrow indicates the >30 Å distance between the ARIH2* UBAL domain and NEDD8. **c**, Left, crystal structure of autoinhibited ARIH2, with domains colored as in Fig. 1a, fit into the cryo-EM map (sharpened by DeepEMhancer^40^) of ARIH2* from complex with neddylated CRL5^Vif-CBFβ^-A3C. Right, crystal structure of autoinhibited ARIH2 with domains colored as in Fig. 1a superimposed with structure of ARIH2* (in gray) from complex with neddylated CRL5^Vif-CBFβ^-A3C. The Rcat domain of ARIH2* is not clearly defined in the density and thus was not modeled. To generate the model for ARIH2*, the coordinates for the UBAL, RING1, RTI-helix, and UBAL domain from the ARIH2 crystal structure were wholesale docked into the cryo EM density, which is relatively lower resolution over this region of the complex. The ARIH2* IBR and Ariadne domains were rebuilt based on the high-quality density for these regions. **d**, Structure of ARIH2* from complex with neddylated CRL5^Vif-CBFβ^-A3C, with domains colored as in Fig. 1a. Structure of corresponding region of ARIH1 (light blue) from a complex with a neddylated CRL1^23^ is shown superimposed. The latter complex represents UB transfer from UBE2L3 to neddylated CRL1-bound ARIH1, but for simplification, the Rcat domain of ARIH1 is not shown. **e**, Structure of E2~UB-binding platform of autoinhibited ARIH2 is shown with domains colored as in Fig. 1a. The corresponding region of the structure representing UB transfer from UBE2L3 to neddylated CRL1-bound ARIH1^23^ was superimposed and is shown in light blue with its bound UBE2L3~UB (cyan and orange). Close-up (rotated 45° in x) shows interface between UBE2L3 and ARIH1 RING1, highlighting the central I188, and corresponding ARIH2* RING1 V141. **f**, Fluorescent scan of gel showing neddylated CRL5^Vif-CBFβ^ and ARIH2-dependent *UB transfer from UBE2L3 through E3-E3 cascade to A3G substrate, comparing WT ARIH2 and V141D mutant. (Source Data Extended Data Fig. 2). The data are representative of N = 2 independent experiments. Source data

We sought cryo-EM data to visualize how neddylated CRL5s bind and activate ARIH2. However, complexes with wild-type (WT) ARIH2 were too heterogeneous to yield high-quality three-dimensional (3D) reconstructions. Assuming that ARIH2 adopts an activated conformation when bound to a neddylated CRL5, we hypothesized that mutationally relieving autoinhibition might improve complex formation. Indeed, the ARIH2* mutant showed enhanced copurification with substrate-bound neddylated CRL5^Vif-CBFβ^ (Extended Data Fig. 2a).

**Extended Data Fig. 3:**
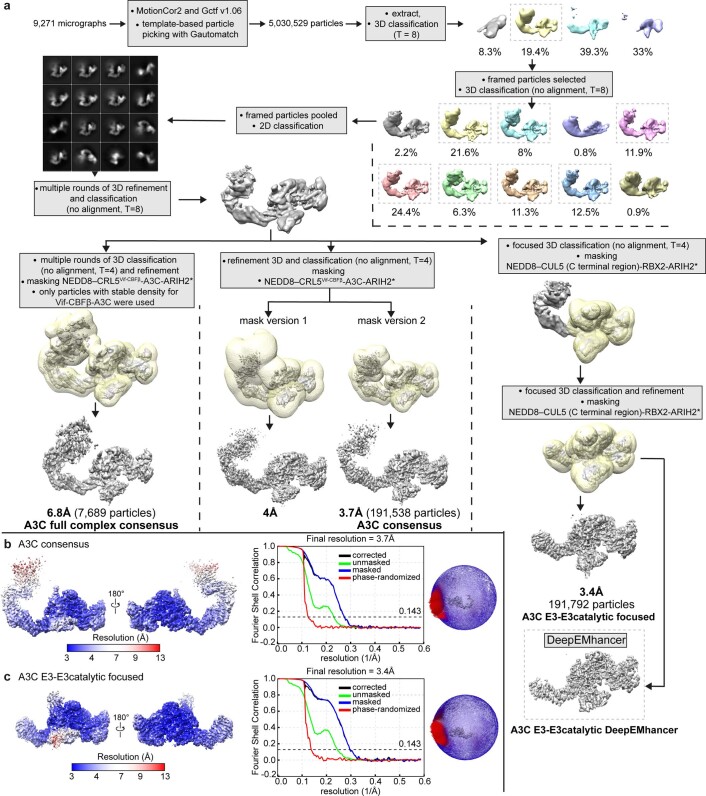
Cryo-EM image processing flowchart for neddylated CRL5^Vif-CBFβ^-ARIH2*-A3C complex. **a**, Cryo-EM image processing flowchart for the complex containing ARIH2*, neddylated CRL5^Vif-CBFβ^, and A3C. Masks are shown in transparent yellow surface superimposed with grey maps. **b**, From left to right: two views showing local resolution colored on the A3C consensus map for the complex containing ARIH2*, neddylated CRL5^Vif-CBFβ^, and A3C; Fourier Shell Correlation (FSC) curve showing the overall resolution of 3.7 Å with the FSC = 0.143 criterion; and angular orientation distribution of the final 3D reconstruction. **c**, As in b, for the focused refined map (A3C E3-E3catalytic focused) over the catalytic assembly containing ARIH2* and a portion of neddylated CUL5 bound to RBX2.

**Extended Data Fig. 4:**
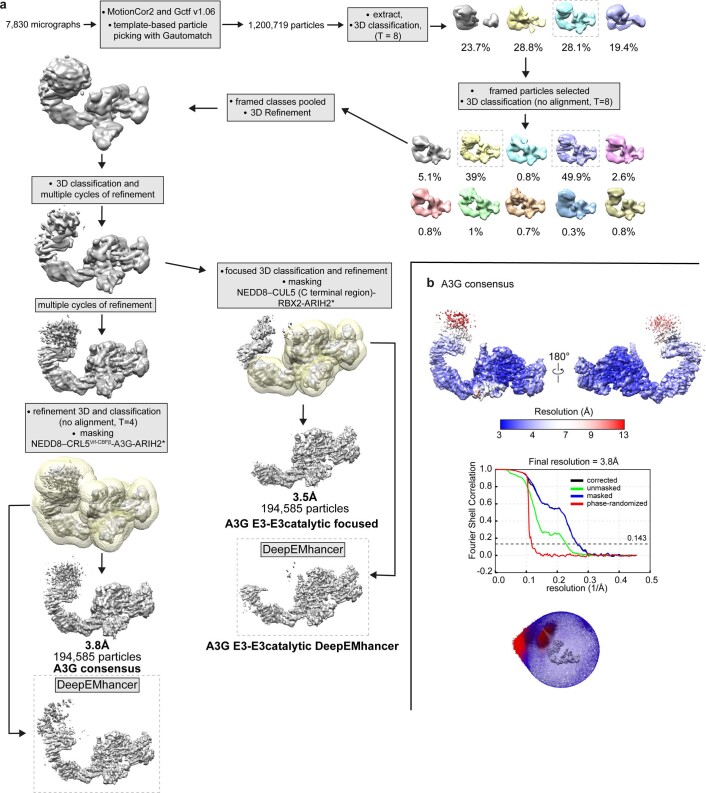
Cryo-EM image processing flowchart for neddylated CRL5^Vif-CBFβ^-ARIH2*-A3G complex. **a**, Cryo-EM image processing flowchart for the complex containing ARIH2*, neddylated CRL5^Vif-CBFβ^, and A3G. The portion of the reconstructions corresponding to A3G-Vif-CBFβ-ELOBC is visible only at low contour. This subcomplex is presumably mobile relative to the assembly between ARIH2* and neddylated CUL5-RBX2. Masks are shown in transparent yellow surface superimposed with grey maps. **b**, From top to bottom: two views showing local resolution colored on the A3G consensus map for the complex containing ARIH2*, neddylated CRL5^Vif-CBFβ^, and A3G; Fourier Shell Correlation (FSC) curve showing the overall resolution of 3.8 Å with the FSC = 0.143 criterion; and angular orientation distribution of the final 3D reconstruction.

**Extended Data Fig. 5:**
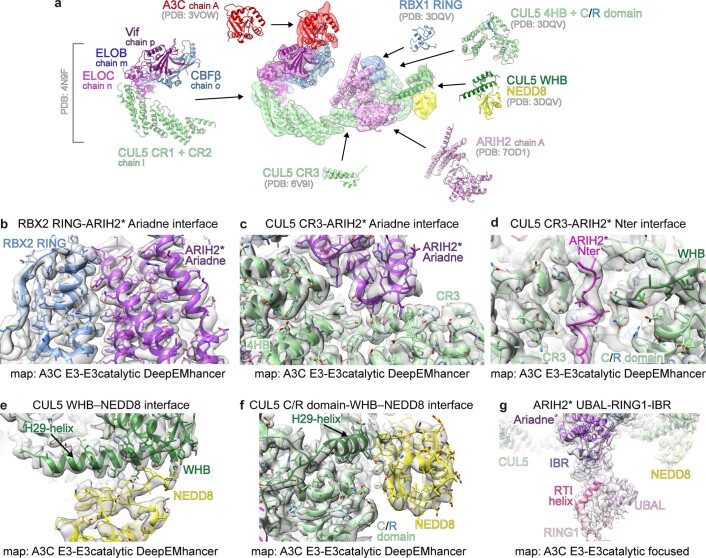
Cryo-EM model building. **a**, Cryo-EM map of ARIH2* complex with neddylated CRL5^Vif-CBFβ^-A3C, low-pass filtered to 7.5 Å resolution, and structures fit into the map^12,22,36,39^. Crystal structure of ARIH2 (PDB ID: 7OD1) was determined in this study. **b**, Close-up of interface between ARIH2* Ariadne domain and RBX2, shown in A3C E3-E3catalytic DeepEMhancer map. **c**, Close-up of interface between ARIH2* Ariadne domain and CUL5, shown in A3C E3-E3catalytic DeepEMhancer map. **d**, Close-up of interface between ARIH2* N-terminal region and CUL5 groove, shown in A3C E3-E3catalytic DeepEMhancer map. **e**, Close-up of interface between NEDD8 and its linked CUL5 H29-helix shown in A3C E3-E3catalytic DeepEMhancer map. **f**, Close-up of 3-way junction between NEDD8 and its linked CUL5 WHB domain, and C/R domain portion of CUL5, shown in A3C E3-E3catalytic DeepEMhancer map. **g**, Domains from crystal structure of autoinhibited ARIH2 shown fit into A3C E3-E3catalytic focused map.

We obtained cryo-EM maps for two ARIH2* complexes with neddylated CRL5^Vif-CBFβ^, one with the substrate APOBEC3C and the other with APOBEC3G (hereafter A3C and A3G, respectively) (Extended Data Figs. 2–4 and Supplementary Table 2). Because both complexes showed similar properties, only the higher resolution reconstructions with A3C are described. A 7.5 Å resolution low-pass filtered map allowed fitting with published atomic coordinates of A3C, Vif-CBFβ-ELOBC bound to CUL5’s N-terminal domain, other domains of CUL5, NEDD8 and nearly all the ARIH2 crystal structure^12,22,36–39^ (Fig. 1c,d and Extended Data Fig. 5). However, lack of density unambiguously attributable to ARIH2*’s Rcat suggests that this domain is relatively mobile compared to the rest of the E3-E3 complex (Extended Data Fig. 2c).

The neddylated CRL5-ARIH2* E3-E3 assembly confirms several previous predictions^5,9,16,23,33^. First, the A3C (or A3G) substrate and ARIH2* are bound at opposite ends of the elongated neddylated CUL5-RBX2 and directed toward each other, presumably to promote catalytic encounter (Fig. 1d). Second, ARIH2*’s UBAL, RING1, RTI helix and IBR elements are configured as in the crystal structure of autoinhibited ARIH2, and superimpose with the corresponding E2~UB-binding platform of ARIH1 bound to a neddylated CRL1 (ref. ^23^) (Extended Data Fig. 2c,d). Mutation of Val141 in ARIH2’s RING1 domain, paralogous to a key ARIH1 RING1 domain residue recruiting E2~UB, impaired ubiquitylation of a neddylated CRL5 substrate, confirming common ARIH-family RBR E3 enzymatic mechanisms (Extended Data Fig. 2e,f).

### NEDD8-dependent allosteric remodeling of CUL5-RBX2

**Fig. 2:**
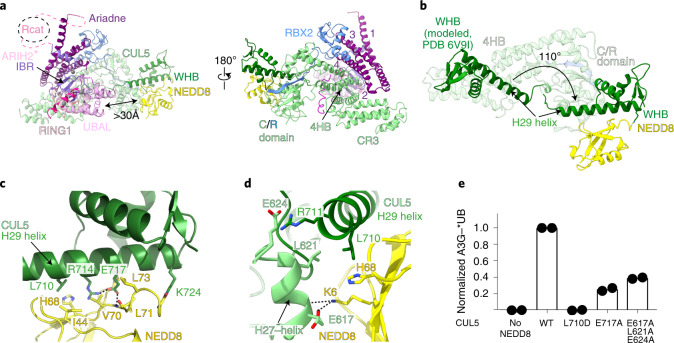
NEDD8 conformational activation of CUL5-RBX2. **a**, Structure of ARIH2* complex with neddylated CUL5 (spanning from CR3 domain to C terminus)-RBX2 is shown with domains colored as in Fig. 1. Black arrow indicates the >30 Å distance between the ARIH2* UBAL domain and NEDD8. Zinc atoms as spheres, and Ariadne domain helices numbered. **b**, Structural transition of CUL5 H29-helix and WHB domain between the unneddylated CUL5-RBX2 and neddylated CRL5^Vif-CBFβ^-A3C-ARIH2* complex (ARIH2* and RBX2 RING domain are not shown for simplification). To visualize the relative position of the unneddylated CUL5 H29-helix and WHB domain (dark green), the region encompassing the CR3, 4HB and C/R domains from unneddylated CUL5-RBX2 (ref. ^22^) was superimposed on the neddylated CUL5-RBX2-ARIH2* structure, but is not shown for simplification. In unneddylated CUL5, the H29-helix encompasses residues 697–725, whereas the first turn is unfolded and the H29-helix encompasses residues 700–725 in neddylated CUL5. **c**, Close-up of interactions surrounding the covalent isopeptide linkage between NEDD8 (yellow) and CUL5 H29-helix portion of the WHB domain (dark green). **d**, Three-way interface between NEDD8, its covalently linked CUL5 H29 helix and WHB domain, and a CUL5 surface from the C/R domain (relative to **c**, rotated 80° in *x* and 40° in *y*). **e**, ARIH2-catalyzed fluorescent UB (*UB) transfer to A3G in 10 min, mediated by WT unneddylated (no NEDD8), WT neddylated CRL5^Vif-CBFβ^ (WT) or versions with indicated mutations of CUL5 residues making noncovalent interactions with NEDD8. *N* = 2 independent experiments. For samples from same experiment, gels were processed in parallel (Source Data Fig. 2). Source data

Focused refinement^40^ yielded a 3.4 Å resolution map allowing generation of atomic coordinates showing interactions between neddylated CUL5-RBX2 and ARIH2* (Fig. 2a and Extended Data Figs. 2b and 3–5). CUL5-linked NEDD8 does not approach ARIH2*. Their closest residues are separated by more than 30 Å (Fig. 2a). NEDD8’s concave β-sheet embraces two domains from CUL5, resulting in a striking approximately 110° rotation of CUL5’s rod-like H29-helix and repositioning of the WHB domain compared to its position in unneddylated CUL5 (refs. ^12,22^) (Fig. 2b–d).

**Extended Data Fig. 6:**
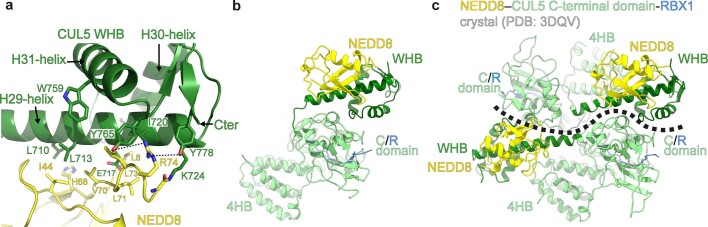
Comparison of NEDD8 interactions with CUL5 in cryo-EM structure of neddylated CUL5-RBX2-ARIH2* and crystal of neddylated CUL5-RBX1. **a**, Close-up of the noncovalent interface between NEDD8 (yellow) and its linked CUL5 WHB domain (green), from the cryo-EM structure of neddylated CRL5^Vif-CBFβ^-A3C complex with ARIH2*. Side-chains mediating contacts, and CUL5 H29-, H30-, and H31-helices are indicated. **b**, Portion of the cryo-EM structure corresponding to NEDD8, CUL5 C-terminal domain (encompassing the 4HB, C/R domain, and WHB domain), and the N-terminal strand of RBX2 contributing to the C/R domain. **c**, Lattice packing in prior crystal structure of neddylated CUL5 C-terminal domain (CTD)-RBX1 complex^12^, highlighting CUL5’s H29-helix and WHB domain in dark green, and its linked NEDD8 in yellow. Dotted line indicates the border between the two different neddylated CUL5 CTD-RBX1 complexes in the crystal lattice. NEDD8 and CUL5 interact similarly as in the cryo-EM structure, although contacts between NEDD8 and its linked CUL5 WHB domain are with the C/R domain of the adjacent complex in the crystal.

NEDD8’s Ile44-centered hydrophobic patch makes extensive noncovalent interactions with CUL5’s WHB domain (Fig. 2c and Extended Data Fig. 6a). NEDD8’s Ile44 and Val70 interact with Leu710 and Leu713 from the WHB domain portion of CUL5’s H29-helix. To one side, NEDD8’s Leu8 is inserted into a hydrophobic pocket between CUL5’s H29 and H30 helices. On the other side, NEDD8’s Leu73 and Arg74 intercalate between CUL5’s Ile720, Trp759, Tyr765, Tyr778, CUL5’s C terminus and the isopeptide bond linking NEDD8 to CUL5’s Lys724. Additionally, CUL5’s Glu717 is poised to coordinate a network of electrostatic interactions with NEDD8 (Extended Data Fig. 6a). Notably, this NEDD8–CUL5 interface was already observed in the crystal of a neddylated CUL5 C-terminal region bound to RBX1 (reported before discovery of neddylation enzymes for CUL5-RBX2)^12^ (Extended Data Fig. 6b,c).

NEDD8 also binds the edge of CUL5 in the intermolecular C/R domain. NEDD8’s Lys6 and His68 form a three-way interface with Leu710 from CUL5’s H29-helix and a stripe of Glu617, Leu621 and Glu624 side-chains from CUL5 in the C/R domain (Fig. 2d). Retrospective analysis revealed the same three-way interactions in the previous crystal of neddylated CUL5’s C-terminal region, albeit with a twist: in the crystal, rather than occurring within a single complex, these interactions mediate packing between the C/R domain from one molecule of CUL5 and NEDD8 and its linked WHB domain from an adjacent complex in the lattice^12^ (Extended Data Fig. 6b,c). Mutation of the key NEDD8 binding surfaces on CUL5’s WHB and C/R domain impaired ARIH2-mediated ubiquitylation of neddylated CRL5 substrates (Fig. 2e).

### ARIH2-CUL5-RBX2 E3-E3act superdomain

**Fig. 3:**
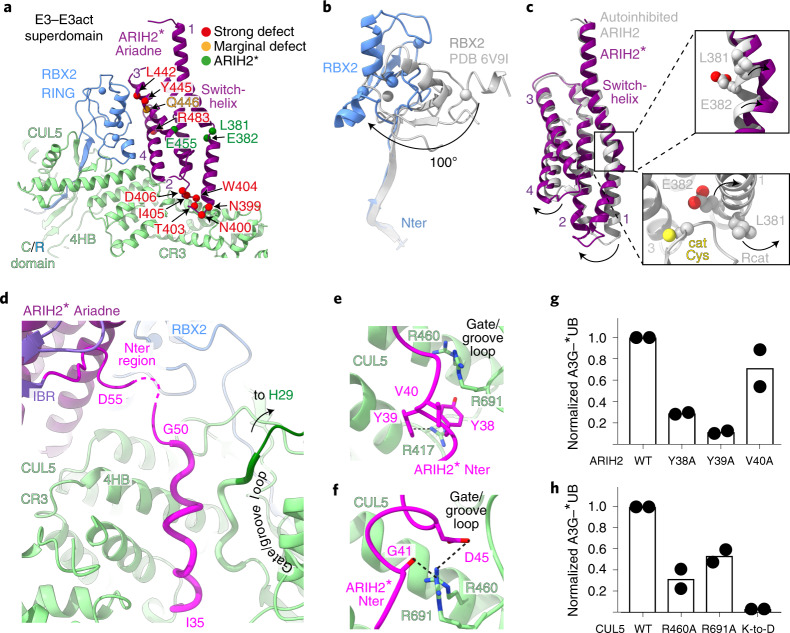
Details of ARIH2*-neddylated CUL5-RBX2 E3-E3 assembly. **a**, Ariadne domain of ARIH2* (purple, helices numbered) binds CUL5 (green) and RBX2 (blue) to form a singular E3-E3act superdomain. Mutations defining ARIH2*, or defective in neddylated CRL5^Vif-CBFβ^-dependent A3G ubiquitylation, are indicated. **b**, Structural transition of RBX2 RING between unneddylated CUL5-RBX2 (white)^22^ and neddylated CRL5^Vif-CBFβ^-A3C-ARIH2* complex (blue). **c**, Structural transition of ARIH2 Ariadne domain (helices numbered) between autoinhibited conformation in crystal structure (white) and ARIH2* complex with neddylated CRL5^Vif-CBFβ^-A3C (purple). The top close-up shows residues at switch-helix kink, which in autoinhibited ARIH2 secure the Rcat domain. The bottom close-up (rotated 85° in *y*) shows ARIH2 Ariadne domain switch-helix residues securing the Rcat domain loop harboring the catalytic Cys in autoinhibited ARIH2. Arrows indicate residue reorientation by switch-helix kink in ARIH2* bound to neddylated CRL5. **d**, Interactions between ARIH2* N-terminal (Nter) region (magenta) and neddylated CUL5 (green) groove generated on reorientation of CUL5’s H29-helix. The remodeled gate/groove loop (residues 691–695) contributes to binding ARIH2*. ARIH2* residues demarking structural elements are indicated. **e**, Close-up of hydrophobic ARIH2* residues binding remodeled neddylated CUL5 groove. **f**, Close-up of CUL5 arginines from across the groove securing ARIH2* N terminus. **g**, Neddylated CRL5^Vif-CBFβ^-dependent *UB transfer to A3G in 10 min, catalyzed by WT ARIH2 or with indicated ARIH2 mutants. *N* = 2 independent experiments. **h**, ARIH2-catalyzed *UB transfer to A3G in 10 min, mediated by WT neddylated CRL5^Vif-CBFβ^ or indicated CUL5 mutants. ‘K-to-D’ is CUL5 K418D K423D K676D K685D. *N* = 2 independent experiments. For samples derived from the same experiment, gels were processed in parallel (Source Data Fig. 3). Source data

**Extended Data Fig. 7:**
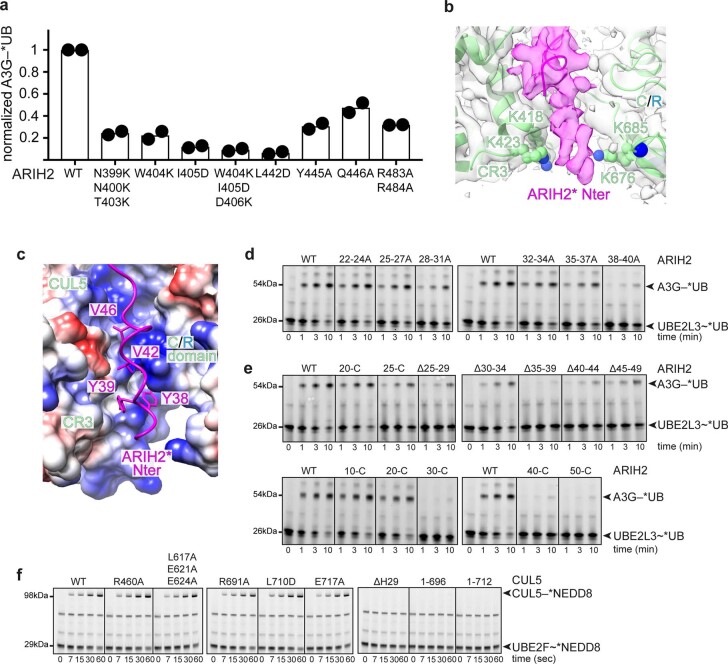
Interactions between ARIH2 N-terminal region and remodeled CUL5 groove. **a**, Neddylated CRL5^Vif-CBFβ^-dependent fluorescent UB (*UB) transfer to A3G in 10 minutes, catalyzed by WT ARIH2 or indicated Ariadne domain mutants. (Source Data Extended Data Fig. 7). N = 2 independent experiments. For samples from same experiment, gels were processed in parallel. **b**, Close-up showing cryo-EM density at low contour, over ARIH2*’s N-terminal region (magenta) and neddylated CRL5 groove. Basic residues from neddylated CUL5 (K418, K423, K676 and K685) line the groove and are poised to contact the largely acidic ARIH2 N-terminal stretch (residues 22-34) not modeled in the structure. **c**, Close-up of cryo-EM structure, showing complementarity between ARIH2* N-terminal region (magenta) and CUL5 groove shown as surface colored by electrostatic potential (red negative, white neutral, blue positive). **d**, Fluorescent scans of gels showing neddylated CRL5^Vif-CBFβ^ and ARIH2-dependent *UB transfer from UBE2L3 through E3-E3 cascade for ubiquitylating A3G substrate over time (Source Data Extended Data Fig. 7). The assay follows the scheme shown in Extended Data Fig. 1d. The assays test effects of indicated multi-Ala mutants in ARIH2 N-terminal domain. Residues substituted with Ala are encompassed by the indicated region, for example 22–24 A is 22 A 23 A 24 A. The data are representative from N = 2 independent experiments. **e**, Assay as b, except with indicated deletion-mutant versions of ARIH2 (Source Data Extended Data Fig. 7). **f**, Chase assays monitoring neddylation of various CUL5-RBX2 mutants as quality control for proper folding. Assays were performed in pulse-chase format, and detect fluorescent *NEDD8. *NEDD8 harbors an N-terminal tag wherein a cysteine is conjugated to a fluorescein-5-maleimide. First, the reactive, thioester-linked UBE2F~*NEDD8 intermediate was generated in a pulse reaction catalyzed by the NEDD8-specific E1 enzyme (the APPBP1-UBA3 complex). After the pulse reaction was quenched, the chase reaction was initiated by adding either WT or indicated mutant versions of CUL5-RBX2, and *NEDD8 transfer from UBE2F to CUL5 was monitored over time. The deletion mutants of CUL5 all lack the neddylation site (K724) and are: ΔH29 lacking residues 693–725, 1–696 lacking residues 697–780, and 1–712 lacking residues 713–780. Shown are fluorescent scans of SDS-PAGE gels of reaction products (Source Data Extended Data Fig. 7). The data are representative from N = 2 independent experiments. Source data

Two surfaces from ARIH2* bind neddylated CUL5-RBX2. One interaction involves ARIH2*’s Ariadne domain binding CUL5 and RBX2 in an E3-E3act superdomain (Fig. 3a). The homologous E3-E3act superdomain formed by ARIH1 and neddylated CUL1-RBX1 was named for its amalgamation of the two distinct types of E3 and activating ubiquitylation^23^. Here the loop between the first and second helices of ARIH2*’s Ariadne domain docks in a cleft from CUL5’s CR3 and 4HB domains. The third and fourth helices, on other side of ARIH2*’s Ariadne domain, bind RBX2’s RING domain and the junction with CUL5. Mutating ARIH2 Ariadne domain residues binding CUL5 and RBX2 impairs substrate ubiquitylation, confirming the importance of the E3-E3act superdomain (Fig. 3a, Extended Data Fig. 7a).

Both E3s undergo conformational changes to form the E3-E3act superdomain (Supplementary Video 1). Relative to its orientation in an unnedddylated CRL5 (ref. ^22^), RBX2’s RING undergoes a roughly 100° rotation to bind the ARIH2* Ariadne domain (Fig. 3b). Also, comparing the Ariadne domain conformations in autoinhibited ARIH2 and ARIH2* bound to CUL5 shows reorientation of the helices (Fig. 3c). In particular, the first Ariadne domain helix, which we term a ‘switch-helix’, displays a roughly central 15° kink when bound to CUL5. ARIH2 residues 380 and 381 (alanines in ARIH2*) at the center of the kink along with nearby side chains are rotated outward. The switch-helix kink precludes autoinhibitory interactions with the Rcat domain’s catalytic Cys (Fig. 3c). Thus, it seems that when ARIH2 is bound to neddylated CUL5-RBX2, kinking of the switch-helix would relieve autoinhibition. This rationalizes the previous finding that neddylated CUL5-RBX2 stimulates reactivity of ARIH2’s catalytic Cys with the electrophilic UB probe, UB-VME^21^.

### Remodeled CUL5 groove cradles ARIH2 N terminus

The second crucial portion of ARIH2 is its N-terminal region, which is not present in the crystal structure. In complex with a neddylated CRL5, ARIH2*’s N terminus mediates interactions extending more than 50 Å across the structurally remodeled CUL5 (Fig. 3d).

The central portion of ARIH2*’s N-terminal region (residues 35–46) forms a kinked amphipathic helix that docks in a CUL5 groove. One side of the groove is formed by CUL5’s 4HB domain. The other side involves CUL5 elements from the C/R domain and the loop preceding its H29-helix, which we term ‘gate/groove loop’ (Fig. 3d). In an unneddylated CRL5 complex, the gate/groove loop (CUL5 residues 691–695) restricts access to CUL5’s 4HB. However, in neddylated CUL5 the H29-helix rotation is accompanied by gate/groove loop remodeling, which generates the ARIH2-binding groove.

Ile35, Tyr38, Tyr39, Val42 and Val46 from ARIH2* form a stripe of hydrophobic knobs that fit into hydrophobic pockets between CUL5 4HB domain helices (Fig. 3e,f and Extended Data Fig. 7b,c). A pair of neddylated CUL5 arginines—Arg460 from the 4HB and Arg691 from the gate/groove loop—seal ARIH2*’s Tyr39 in the groove. CUL5’s Arg691 additionally interacts with the backbone carbonyl from ARIH2*’s Gly41 at the kink and Asp45 at the C terminus of the helix (Fig. 3f).

Density was poorly visible for the elements connected to ARIH2*’s N-terminal region. Residues 50–55 comprise a linker to the canonical RBR elements (Fig. 3d). At the opposite, extreme N-terminal end, additional density was visible only at low contour, in a basic groove from CUL5 (Extended Data Fig. 7b).

To interrogate roles of ARIH2’s N-terminal region, we performed alanine scanning mutagenesis. Substituting residues individually, or three or four at a time, confirmed key roles of the region containing ARIH2’s Tyr38 and Tyr39, and intermediate effects at the junction to the acidic stretch (Fig. 3g and Extended Data Fig. 7d). We also tested effects of deletions (Extended Data Fig. 7e). The most destructive effects arose from removing portions of ARIH2’s N-terminal region—either residues 35–39 or 40–44—that dock in the remodeled CUL5 groove. Deleting the N-terminal 20 residues, which were not observed by cryo-EM, did not overtly impair ubiquitylation. However, deletions within an ARIH2 acidic stretch (residues 25–29 or 30–34) impaired ubiquitylation of neddylated CRL5 substrates. We speculate that the ARIH2 acidic residues could contact basic residues at the entrance to the CUL5 groove. Notably, Ala substitution for either CUL5 Arg460 or Arg691, or four basic residues at the edge of the CUL5 groove (Lys418, Lys423, Lys676 and Lys685) substantially impair ARIH2-mediated ubiquitylation of CRL5^Vif-CBFβ^ substrates (Fig. 3h). None of the mutations impaired CUL5 neddylation, suggesting they did not affect protein folding (Extended Data Fig. 7f).

### CRL5 neddylation removes barriers blocking ARIH2

**Fig. 4:**
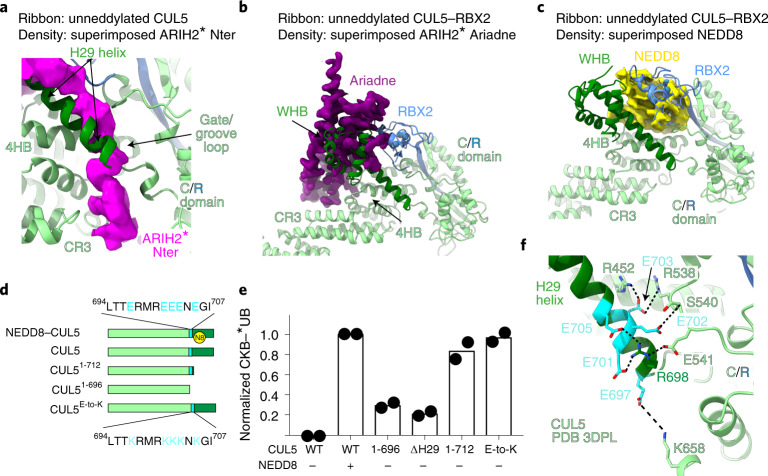
ARIH2 binds surfaces exposed by NEDD8-mediated CUL5-RBX2 conformational changes. **a**, ARIH2’s N-terminal region binds a groove that in unneddylated CUL5-RBX2 is occupied by CUL5’s gate/groove loop and H29-helix, shown by overlaying cryo-EM density for ARIH2* N-terminal region (magenta) from the complex with neddylated CRL5^Vif-CBFβ^-A3C on unneddylated CUL5 (green)-RBX2 (ref. ^22^) aligned over the C/R domain. **b**, ARIH2’s Ariadne domain binds a surface that in unneddylated CUL5-RBX2 is occupied by CUL5’s WHB and RBX2’s RING domains, shown by overlaying cryo-EM density for ARIH2* Ariadne domain (purple) from the complex with neddylated CRL5^Vif-CBFβ^-A3C on unneddylated CUL5-RBX2 (green and blue)^22^ aligned over the C/R domain. **c**, CUL5-linked NEDD8 binds a surface that in unneddylated CUL5-RBX2 is occupied by RBX2’s RING domain, shown by overlaying cryo-EM density for NEDD8 (yellow) linked to CUL5 in complex with ARIH2* on WHB domain of unneddylated CUL5-RBX2 (green and blue)^22^. **d**, Schematics of CUL5 constructs used to test effects of removing regions of unneddylated CUL5 shown in **a** and **b** as blocking ARIH2-binding sites. CUL5’s H29-helix and WHB domain are indicated in dark green. Glutamates securing position of the unneddylated CUL5 H29-helix are indicated in cyan in WT CUL5. Lysine substitutions in CUL5 ‘E-to-K’ mutant are shown below. **e**, ARIH2-catalyzed fluorescent UB (*UB) transfer to CKB in 10 min, mediated by unneddylated or neddylated WT CRL5^ASB9^, or with indicated mutant versions of unneddylated CUL5. *N* = 2 independent experiments. For samples derived from the same experiment, gels were processed in parallel (Source Data Fig. 4). **f**, Structure of unneddylated CUL5 (ref. ^12^) showing interaction network of glutamates in H29-helix. Source data

Although CUL5-linked NEDD8 does not directly bind ARIH2*, neddylation structurally activates the E3-E3 assembly in several ways. Gate/groove loop remodeling not only provides a binding site for ARIH2’s N-terminal region, but also eliminates blockage of the groove (Fig. 4a). Also, when unneddylated, the WHB domain packs against CUL5’s 4HB and RBX2’s RING domains so as to block access of ARIH2’s Ariadne domain (Fig. 4b). This unneddylated arrangement is also incompatible with noncovalent interactions between CUL5’s WHB domain and NEDD8 (Fig. 4c)^12,22^. Considering that the neddylation reaction requires yet another distinct relative arrangement of RBX RING and cullin WHB domains^41^, we speculate that after NEDD8 linkage to CUL5, formation of the structurally observed noncovalent interactions hinders these domains from adopting the orientations in unneddylated CRL5.

The structurally observed conformational changes explain previously reported HDX properties of an unneddylated and neddylated CRL5, ARIH2, and the neddylated CRL5-ARIH2 complex^33^. Deuterium incorporation was measured by mass spectrometry of peptides generated after HDX was quenched^33^. Peptides corresponding to several regions of neddylated CRL5 and ARIH2 remodeled in the cryo-EM structure, for example NEDD8 and the regions of CUL5 it binds, were not detected by mass spectrometry^33^. Nonetheless, the detectable regions that showed greatest HDX differences upon complex formation correlate with the conformational changes indicated by the cryo-EM structure (Supplementary Video 1). In particular, the HDX differences^33^ between unneddylated and neddylated CRL5 primarily map to CUL5 4HB and C/R domain regions exposed by the structurally observed relocation of neddylated CUL5’s WHB domain. Comparing HDX properties of ARIH2 alone versus bound to a neddylated CRL5 showed greatest differences in the Ariadne domain^33^. The regions showing increased HDX correspond to the switch- and subsequent Ariadne domain helices that become exposed in the structural transition between autoinhibited ARIH2 and neddylated CRL5^Vif-CBFβ^-bound ARIH2*. Meanwhile, Ariadne domain regions that were protected from HDX in the complex^33^ correspond to ARIH2* elements that bind CUL5-RBX2. Likewise, the CUL5 regions whose HDX properties differ in the complex with ARIH2 (ref. ^33^) correspond to those bound to ARIH2* in the cryo-EM structure.

The ultimate test of NEDD8’s allosteric role would be to mutationally elicit such activation. Thus, we wondered if removing CUL5’s WHB domain and/or the H29-helix, would be sufficient to activate ARIH2 ubiquitylation of a CRL5 substrate. Such deletion mutants would in principle remove the barrier blocking ARIH2’s Ariadne domain, although they would preclude interactions that stabilize the remodeled the gate/groove loop. The deletions did increase ARIH2-mediated ubiquitylation of CKB compared to unneddylated CRL5^ASB9^, although not to the level observed with neddylation (Fig. 4d,e). We thus inspected the structures of unneddylated^12^ and neddylated CUL5 to identify residues potentially anchoring the inactive conformation, but whose mutation would not hinder relocation of the H29-helix. In unneddylated CUL5, four CUL5 H29-helix glutamates (Glu701, Glu702, Glu703, Glu705) either directly contact the C/R domain or establish H29-helix-C/R domain electrostatic networks (Fig. 4f). A flanking glutamate (Glu697) also may contribute to the inactive conformation. All five of these H29-helix glutamates are solvent-exposed—their side chains not visible in the maps—in neddylated CRL5 bound to ARIH2*. Thus, we hypothesized that charge-swap mutants could expunge unneddylated CUL5’s H29-helix and WHB domain, while allowing the active conformation. Indeed, an ‘E-to-K’ mutant version of unneddylated CRL5^ASB9^ with these CUL5 H29-helix glutamates replaced with lysines enabled ARIH2-dependent CKB ubiquitylation at a level similar to that achieved with neddylated CRL5^ASB9^ in our assay (Fig. 4e).

### General and specific neddylated CRL-ARIH E3-E3 features

**Extended Data Fig. 8:**
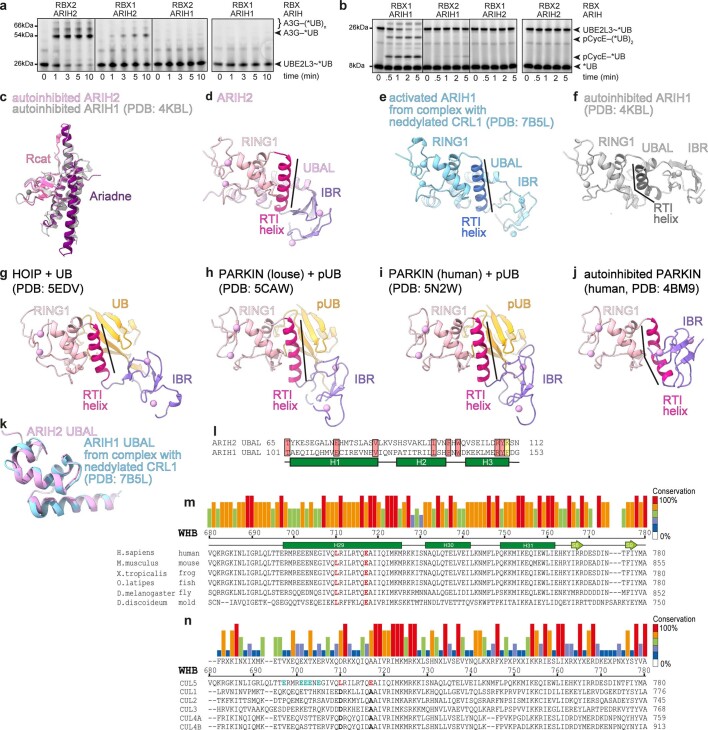
Neddylated CUL5-RBX2-ARIH2 and neddylated CUL1-RBX1-ARIH1 specificity. **a**, Fluorescent scan of gels showing neddylated CRL5^Vif-CBFβ^ and ARIH-family RBR E3-dependent *UB transfer from UBE2L3 through E3-E3 cascade for ubiquitylating A3G substrate, testing roles of ARIH E3 identity (ARIH1 or ARIH2) and RBX identity in neddylated CRL5^Vif-CBFβ^ generated from Vif-CBFβ-ELOBC and either neddylated CUL5-RBX2 or CUL5-RBX1 (Source Data Extended Data Fig. 8). The data are representative from N = 2 independent experiments. **b**, Assays performed as in a, except with neddylated CRL1^FBXW7∆D^ and phosphopeptide derived from Cyclin E (pCycE) as substrate (Source Data Extended Data Fig. 8). **c**, Superposition of Ariadne and Rcat domains in autoinhibited ARIH2 and ARIH1 (PDB ID: 4KBL^25^). **d**, UBAL–RING1–RTI-helix–IBR domains (that is, E2~UB-binding platform) of ARIH2 crystal structure. **e-j**, RING1–RTI-helix–IBR domains (that is, E2~UB-binding platform) of different RBRs (neddylated CRL1 bound ARIH1 PDB ID: 7B5L^23^, autoinhibited ARIH1 PDB ID: 4KBL^25^, HOIP PDB ID: 5EDV^34^, louse PARKIN PDB ID: 5CAW^42^, human PARKIN PDB ID: 5N2W^43^, autoinhibited human PARKIN PDB ID: 4BM9^26^) aligned over their RING1 domains as in **d**. **k**, Superposition of the UBAL domain of ARIH2 crystal structure with that of a neddylated CRL1-bound ARIH1 (PDB ID: 7B5L^23^). **l**, Alignment of UBAL domain sequences of human ARIH2 and ARIH1. Secondary structures are indicated by rectangles for helices. Residues identical between the two are shaded in rose. ARIH1 F150 and ARIH2 K110 are shaded in yellow. **m**, Alignment of WHB domain sequences for CUL5 from the indicated organisms. Secondary structures based on crystal structure of unneddylated CUL5^12^ are indicated by rectangles for helices, arrows for β-strands. Degree of conservation is indicated by color-coded bars above. L710 and E717, which configure noncovalent interactions with covalently linked NEDD8 are highlighted in red. **n**, Alignment of WHB domain sequences from human CUL5, CUL1, CUL2, CUL3, CUL4A, and CUL4B. Degree of conservation is indicated by color-coded bars above. In CUL5 sequence, L710 and E717, which configure noncovalent interactions with covalently linked NEDD8 are highlighted in red. The corresponding aspartate and alanine residues from CULs1–4 are highlighted black. CUL5 glutamates that are candidates for securing H29-helix in unneddylated CUL5 and that were mutated to lysines in E-to-K mutant are highlighted in cyan. Source data

We confirmed and extended previous findings that CRL-ARIH pairing is strikingly specific. Neither ARIH1 nor ARIH2 was active with a noncognate neddylated CRL^9,16,21^, nor with alternative versions harboring mismatched CUL1-RBX2 or CUL5-RBX1 core scaffolds (Extended Data Fig. 8a,b).

**Fig. 5:**
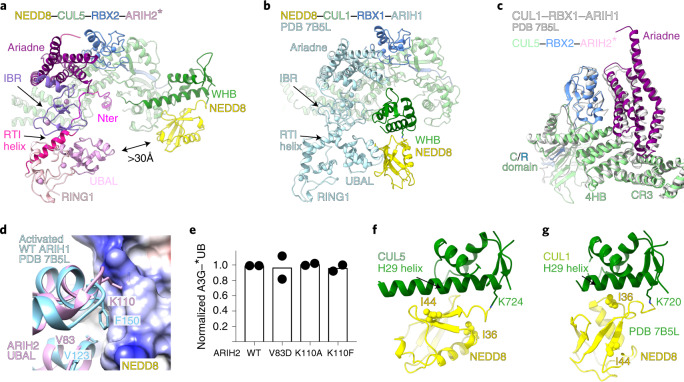
Comparison of neddylated CRL5-ARIH2 and neddylated CRL1-ARIH1 E3-E3 assemblies. **a**, Cryo-EM structure of neddylated CUL5-RBX2-ARIH2* complex with domains colored according to Fig. 1. NEDD8, isopeptide-bonded to CUL5, is positioned more than 30 Å away from the nearest ARIH2* residue. **b**, Neddylated CUL1-RBX1-ARIH1 assembly from the previous cryo-EM structure of chemically trapped complex representing UB transfer from UBE2L3 to ARIH1 (other regions of structure not shown)^23^. NEDD8 (yellow), isopeptide-bonded to CUL1’s WHB domain, directly binds ARIH1’s (light blue) UBAL domain. **c**, Superposition of intermolecular E3-E3act superdomains from CUL5-RBX2-ARIH2* (colored) and CUL1-RBX1-ARIH1 (white)^23^. **d**, Superposition of UBAL domain of ARIH2 (pink) with that of ARIH1 (blue) bound to NEDD8 (colored by electrostatic surface: red negative, blue positive, and white uncharged) from the complex with a neddylated CRL1 (ref. ^23^). **e**, Neddylated CRL5^Vif-CBFβ^-dependent fluorescent UB (*UB) transfer to A3G in 10 min, catalyzed by WT ARIH2 or indicated UBAL domain mutants. *N* = 2 independent experiments. For samples derived from the same experiment, gels were processed in parallel (Source Data Fig. 5). **f**, Interactions between NEDD8 (yellow) and its linked CUL5 WHB domain (dark green). NEDD8 I36 and I44 side chains are shown as spheres. **g**, Interactions between NEDD8 (yellow) and its linked CUL1 WHB domain (dark green)^23^. NEDD8 I36 and I44 side chains are shown as spheres. Source data

To gain further insights into similarities and differences between E3-E3 ligases, we compared structures of ARIH2 and ARIH1, and their complexes with a neddylated CRL5 or CRL1, respectively (Fig. 5a,b). The comparison showed similar roles of the ARIH2 and ARIH1 Ariadne domains. The Ariadne-Rcat domain arrangements superimpose in autoinhibited ARIH2 and ARIH1 (0.8 Å r.m.s.d., Extended Data Fig. 8c), and the E3-E3act domains also superimpose for both families (1.1 Å r.m.s.d., Fig. 5c). Notably, the Ariadne domain switch-helix kink observed for ARIH2* is shared by both WT ARIH1 and the corresponding ARIH1* mutant when bound to the cognate neddylated CRL^23^.

However, the two E3-E3 pathways show substantial differences in interactions directly affected by neddylation. In autoinhibited ARIH2, the RING1, RTI helix and IBR domains are arranged much like in activated, E2~UB-bound ARIH1 and other RBR E3s^35,42–45^ (Extended Data Fig. 8d–j). However, these E2~UB-binding platform elements are misaligned in autoinhibited ARIH1 (refs. ^23,25,46,47^). These elements are also misaligned in autoinhibited PARKIN^26–28^.

Activation of ARIH1 depends on Val123 and Phe150 in its UBAL domain binding the Ile44-centered hydrophobic patch in CUL1-linked NEDD8 (refs. ^16,21,23^). However, despite sharing a common fold, the sequence of ARIH2’s UBAL domain is divergent. Notably, Lys110 corresponding to ARIH1’s Phe150 is incompatible with hydrophobic interactions (Fig. 5d and Extended Data Fig. 8k,l). Accordingly, ARIH2’s UBAL domain does not bind NEDD8. Mutation of ARIH2 Val83 and Lys110—corresponding to ARIH1’s Val123 and Phe150—did not affect ubiquitylation of neddylated CRL5 substrates (Fig. 5e).

NEDD8 also makes distinct noncovalent interactions with covalently linked CUL5 and CUL1. NEDD8 allosterically activates a CRL5 through its Ile44-centered hydrophobic patch simultaneously packing against CUL5’s H29-helix and C/R domain (Figs. 2 and 5f). However, CUL1’s H29-helix binds a different, Ile36-centered NEDD8 hydrophobic patch^17,23^ (Fig. 5g). The exposed Ile44-centered hydrophobic patch of CUL1-linked NEDD8 binds UB-carrying enzymes including ARIH1 (refs. ^17,23^) (Fig. 5b). The different interactions with NEDD8 are rationalized by the cullin sequences (Extended Data Fig. 8m,n). CUL5’s Leu710 and Glu717 that bind NEDD8’s Ile44 patch would clash with NEDD8’s Ile36 patch. Meanwhile, the corresponding CUL1 residues Asp and Ala, respectively, are conserved across CULs 1–4 and incompatible with NEDD8’s Ile44 patch, explaining the deleterious effects of their swapping into CUL5 (Fig. 2e).

## Discussion

Structures of ARIH2 alone and bound to neddylated CRL5^Vif-CBFβ^ reveal the HIV-1 hijacked E3-E3 ligase assembly that overcomes host restriction and defines mechanisms by which NEDD8-linked CUL5-RBX2 activates ARIH2. Due to high sequence and functional homology, we anticipated that neddylated CRL5-ARIH2 and neddylated CRL1-ARIH1 would form superimposable but sequence-specific E3-E3 assemblies. Indeed, for both, CRL5s and CRL1s, the neddylated conformations remove barriers that mask ARIH E3-binding sites in their unneddylated counterparts. Moreover, for both ARIH2 and ARIH1, the Ariadne domains mediate homologous autoinhibitory interactions with the Rcat domains, and homologous interactions with their cognate CUL-RBX partners.

**Fig. 6:**
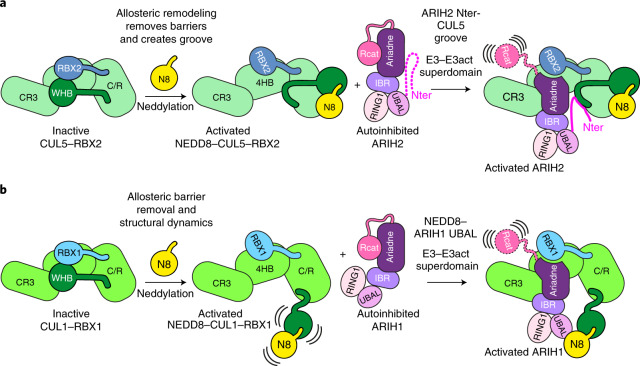
Model of unique NEDD8-driven CRL-ARIH E3-E3 assemblies. **a**, NEDD8 modification of a CRL5 E3 drives assembly with the ARIH2 RBR E3 entirely indirectly, via allostery. Neddylation promotes CUL5-RBX2 conformational changes that remove barriers against and create new binding sites for ARIH2. **b**, NEDD8 distinctly activates CRL1 E3 assembly with the ARIH1 RBR E3 (ref. ^23^). The relative position of NEDD8’s linked CUL1 WHB domain, and arrangement between NEDD8 and CUL1 differ, such that NEDD8 directly recruits ARIH1.

Unexpectedly, however, comparing structures of the homologous E3s—ARIH2 versus ARIH1, and a neddylated CRL5 versus CRL1—individually or in E3–E3 complexes also revealed striking differences, most notably, cullin-specific regulation by NEDD8 (Fig. 6). Different surfaces of NEDD8 interact with covalently linked CUL5 or CUL1 (Fig. 5f,g). CUL1-linked NEDD8 binds directly to ARIH1’s UBAL domain and elicits the activated conformation of the E2~UB-binding platform, a configuration already largely observed in autoinhibited ARIH2 (Figs. 1b and 5a,b). Instead, it is the restructured conformation of the neddylated CRL5, rather than NEDD8 itself, that is recognized by ARIH2. NEDD8 allosterically generates ARIH2-binding surfaces not present in an unmodified CRL5 (Fig. 6).

Why might NEDD8 uniquely modulate the structure of CUL5-RBX2 and its interactions with ARIH2? Although answering this will require future studies, we speculate that additional regulation co-evolved with emergence of CUL5-RBX2 in metazoan lineages. For example, CUL5-RBX2 and/or ARIH2-specific metazoan-specific posttranslational modifications or binding partners awaiting discovery may require a distinctive assembly from that formed by ARIH1 and neddylated CRL1s.

The indirect, allosteric mechanism by which NEDD8 stimulates binding to ARIH2 differs from most characterized interactions between UB and UBLs and their downstream recognition machineries. UB-, SUMO- and LC3-interacting motifs in different proteins often form structurally superimposable complexes with their UB or UBL partners^1,2^. Moreover, to our knowledge, UBL (or UB)-driven protein–protein interactions mediated by conformational changes—without direct binding to the UB or UBL itself—have not been structurally defined before. However, SUMO and UB have been shown to induce conformational changes that inhibit interactions of their targets. For example, a SUMO-interacting motif in thymine DNA glycosylase interacts with a linked SUMO to stabilize a conformation incompatible with DNA-binding^48^. Also, UB-binding domains of the yeast transcription factor Met4 engage a K48-linked polyUB chain modification so as to counteract interactions required to activate transcriptional targets^49^. Such unique allosteric switches, as revealed by our structural analyses, may provide opportunities for therapeutic targeting specificity distinguishing otherwise homologous complexes. This may be particularly relevant for CRL5s and ARIH2, which regulate immune pathways, and are conscripted by several viruses to promote infection^29–32,50^.

## Methods

### Cloning, protein expression and purification

For all expression constructs described in this study, standard molecular biology techniques were used for preparation and verification. Except for HIV-1 Vif (viral infectivity factor), coding sequences of the described proteins are of human origin. Mutant versions of ARIH2, CUL5 and UBE2L3 were generated using the Quikchange system (Agilent) and verified by sequencing.

Open reading frames encoding CUL5 (untagged), RBX2 (a glutathione *S*-transferase (GST) fusion with an intervening tobacco etch virus (TEV) protease site and encompassing RBX2 residues 5 to the C terminus), UBA1 (a GST fusion with an intervening TEV protease site), APOBEC3C (a GST fusion with an intervening TEV protease site) and APOBEC3G (a GST fusion with an intervening TEV protease site) were subcloned into pLIB vectors for expression in *Trichoplusia ni* High-Five insect cells. CUL5 and RBX2 were coexpressed via baculoviral co-infection in a manner similar to that described previously for CUL1-RBX1 (refs. ^17,23^). Briefly, CUL5-RBX2 and UBA1 were initially purified by GST-affinity chromatography, subjected to TEV protease cleavage of the fusions overnight at 4 °C, and further purified by anion exchange chromatography using a HiTrap Q HP column (Cytiva Life Sciences) and then by size-exclusion chromatography. CUL5-RBX2 variants were purified using the same procedure, and are indicated by the residue numbers mutated and/or the ranges encompassed with the following exceptions: the CUL5 mutant ‘Δ29 helix’ lacks residues 694–726; ‘K-to-D’ is K418D K423D K676D K685D; ‘E-to-K’ is E697K E701K E702K E703K E705K. APOBEC3C and APOBEC3G (hereafter referred to as A3C and A3G, respectively) were purified as previously described^37^. Neddylated CUL1-RBX1, SKP1-FBXW7 (the ∆D version lacking the dimerization domain), and ARIH1 were expressed and purified as previously described^16,17,23^. The buffer used for the final size-exclusion chromatography purification of all these proteins and complexes was 25 mM HEPES pH 7.5, 150 mM NaCl and 1 mM DTT. Purity of all protein samples was verified by intact mass spectrometry provided by the Max Planck Institute of Biochemistry Core Facility.

Two base versions of ARIH2 are used. A near full-length version lacking only the first 50 residues (encompassing residues 51 to the C terminus) was used for obtaining the crystal structure of autoinhibited ARIH2. All biochemical assays and cryo-EM studies used full-length versions of either WT ARIH2 or the mutant versions of the full-length construct. ARIH2* refers to a mutant version of full-length ARIH2, relieved from autoinhibition through three residue substitutions: L381A E382A E455A. Deletion mutant versions of ARIH2 are indicated by ‘Δ’ followed by residues excluded from the construct. All versions of ARIH2 were expressed using a common protocol. The constructs, in pRSF vector, contain an N-terminal His_6_-tag followed by maltose binding protein and a TEV protease cleavage site fused to the N terminus of ARIH2 (or residue 51 in the version used in the crystal structure). The various versions of ARIH2 were expressed in in *Escherichia coli* (Rosetta, DE3). Cultures were grown to an optical density of 0.6–0.8 on which expression was induced with 0.1 mM isopropyl-β-d-thiogalactopyranoside (Sigma) and 0.1 mM ZnCl_2_ (Sigma). The various versions of ARIH2 were initially purified by nickel-affinity chromatography, subjected to TEV protease cleavage of the fusions overnight at 4 °C and further purified by anion exchange chromatography using a HiTrap Q HP column (Cytiva Life Sciences) and then by size-exclusion chromatography in 25 mM HEPES pH 7.5, 150 mM NaCl and 1 mM DTT.

An open reading frame encoding HIV-1 Vif (UniProt sequence P12504), codon-optimized for expression in *E. coli*, was obtained from GeneArt/Thermo Fisher. This and the gene encoding human CBFβ were subcloned into pRSF duet vector (MCS1 and MCS2, respectively). Vif was expressed with an N-terminal His_6_-tag followed by a TEV protease cleavage site. Full-length untagged Elongin B (ELOB) and Elongin C (ELOC) were subcloned into MCS1 and MCS2 of pACYCDuet-1 (Novagen), respectively. The Vif, CBFβ and ELOB and ELOC expression plasmids were cotransformed into *E. coli* (BL21 Gold, DE3) and the proteins coexpressed and purified as previously described^37^. The ASB9 complex with ELOBC was expressed similarly, except ASB9 was subcloned into the pET3a vector with an N-terminal His_6_-tag followed by a TEV protease cleavage site. These complexes were initially purified by nickel-affinity chromatography, subjected to TEV protease cleavage of the fusions overnight at 4 °C, and further purified by ion exchange chromatography and then by size-exclusion chromatography in 25 mM HEPES pH 7.5, 150 mM NaCl and 1 mM DTT.

CKB was expressed as an N-terminal GST fusion—with a TEV protease between GST and CKB—in *E. coli* (Rosetta, DE3) cells. Neddylation components NEDD8, UBE2F, UBE2M and APPBP1-UBA3 were expressed in either *E. coli* (Rosetta, DE3) or BL21 Gold, DE3) cells as GST fusions with either thrombin or TEV as intervening protease cleavage sites. These proteins were expressed and purified as previously described^16,41^, with the exception of APPBP1-UBA3, where all fractions containing the neddylation E1 enzyme were pooled to maximize yield rather than purity. The CUL5-RBX2 complex was neddylated by mixing 12 µM CUL5-RBX2, 1 µM UBE2F, 0.2 µM APPBP1-UBA3, 25 µM NEDD8 in 25 mM HEPES pH 7.5, 150 mM NaCl, 10 mM MgCl_2_ and 1 mM ATP. NEDD8 was added at room temperature. Neddylation was quenched after 8 min by adding 10 mM DTT to suppress activity of APPBP1-UBA3 and UBE2F. After microcentrifugation at 13,000 r.p.m. for 10 min, the NEDD8–CUL5-RBX2 was purified using a Superdex SD200 column, in 25 mM HEPES pH 7.5, 150 mM NaCl, 1 mM DTT (– refers to the isopeptide linkage between NEDD8 or UB and a lysine on the target, here a cullin).

*UB refers to human ubiquitin expressed from pGEX-2TK, but with the N-terminal RRASV sequence replaced with RRACV, where the Cys serves as the site for fluorescent labeling with fluorescein maleimide. *UB was expressed in *E. coli* (BL21 RIL, DE3), purified and fluorescently labeled as previously described^41^.

### Peptide

The peptide used as substrate in ubiquitylation assays corresponds to phosphorylated Cyclin E (pCycE) and has sequence KAMLSEQNRASPLPSGLL(pT)PPQ(pS)GRRASY. The peptide was synthesized in the Max Planck Institute of Biochemistry Core Facility, and purified to greater than 95% purify by high-performance liquid chromatography.

### In vitro ubiquitylation assays

*UB transfer was monitored using a pulse-chase format. Briefly, the thioester-bonded UBE2L3~*UB intermediate (the ‘~’ refers to thioester linkage between two proteins) was produced in the pulse reaction, and various proteins were added to initiate the chase reaction in which *UB is transferred from UBE2L3 through the ARIH2-dependent ubiquitylation cascade. Pulse reaction conditions were optimized such that when examined by Coommassie-stained SDS–PAGE, all visibly detectable UBE2L3 was thioester-bonded to UB. The pulse reaction producing UBE2L3~*UB was carried out by incubating 15 μM UBE2L3, 0.3 μM UBA1 and 15 μM *UB in 25 mM HEPES pH 7.5, 100 mM NaCl, 2.5 mM MgCl_2_, 1 mM ATP at room temperature for 30 min. The reaction was quenched with 2 U ml^−1^ apyrase and incubated on ice for at least 5 min. The quenched solution was further diluted to 5 µM with 25 mM HEPES pH 7.5, 100 mM NaCl.

The chase reaction was initiated by adding a premade mixture of various components to the pulse reaction. Chase reaction mixes are described here by the mixture of components, but in final concentrations in the reactions, after addition to the pulse mix. To examine CRL5 activities, the concentration of UBE2L3~*UB generated in the pulse reaction was 0.4 µM. To examine CRL5 substrate ubiquitylation, the chase reaction mix consisted of 0.1 µM ARIH2, 4 µM substrate (A3G, A3C or CKB as indicated), and a neddylated CRL5 assembled in the mix from two parts: 0.4 µM NEDD8–CUL5-RBX2, and either Vif-CBFβ-ELOBC or ASB9-ELOBC as indicated. To examine autoubiquitylation, the chase reaction mix contained 0.4 µM ARIH2 and 0.4 µM NEDD8–CUL5-RBX2 or CRL5. To examine CRL1 activities, the substrate was a phosphopeptide derived from Cyclin E (pCycE, sequence provided above). The chase reaction mix consisted of 0.4 µM UBE2L3~*UB, 2 µM pCycE and 0.5 µM each of ARIH1, NEDD8–CUL1-RBX1 and SKP1FBXW7∆D.

All reactions were carried out at room temperature and quenched at the indicated time points by adding 2× nonreducing SDS–PAGE sample buffer. SDS–PAGE was performed under nonreducing conditions. Gels were scanned using an Amersham Typhoon imager (GE Healthcare). Graphs shown in the main and extended data figures were generated from the 10 min timepoint. Band intensities for ubiquitylated products (*UB linked to A3G or CKB) were measured by ImageQuant TL v.8.2.0.0 and normalized relative to intensities for products generated with WT CUL5 and ARIH2. For samples derived from the same experiment, gels were processed in parallel. Data were processed in Microsoft Excel v.16.16.25 and data points plotted in GraphPad Prism v.8.4.1 (GraphPad Software). All reactions were performed as technical duplicates. All proteins used in assays were roughly 95% pure as judged by Coommassie-stained SDS–PAGE, and molecular weights confirmed by mass spectrometry. For fluorescent ubiquitin, and for ubiquitin-linked proteins, electrophoretic migration was determined by SDS–PAGE and detection by Coommassie staining and fluorescence scanning of the same gel. On this basis, *UB, UBE2L3~*UB, A3G~*UB, CKB~*UB and ARIH2~*UB served as markers of molecular weights 8, 26, 54, 56 and 66 kDa, respectively. The source data files contain the uncropped gel images.

### In vitro neddylation assay

Neddylation of CUL5-RBX2 variants was monitored by using a previously described pulse-chase format^41^, except with UBE2F as E2, RBX2 as E3 and CUL5 as substrate. For the chase reaction, the final concentration of UBE2F~*NEDD8 was 0.2 µM, and CUL5-RBX2 (or indicated variant) was 0.5 µM. Reactions were performed at room temperature in 25 mM HEPES, 100 mM NaCl pH 7.5. Samples were taken the indicated time points, quenched with nonreducing 2× SDS–PAGE sample buffer, run on SDS–PAGE and scanned with an Amersham Typhoon imager (GE Healthcare).

### Crystallization of autoinhibited ARIH2

The N-terminal 50 residues of ARIH2 are predicted to be disordered^21^ and were not included in the version used for crystallization. Crystals of ARIH2 (a version encompassing residues 51 to the C terminus) were grown at 4 °C by the sitting drop vapor diffusion method. Then 10 mg ml^−1^ protein was mixed in a 1:1 ratio with 0.2 M sodium nitrate, 0.1 M Bis-Tris propane pH 8.5 and 20% PEG 3350 at 4 °C. Crystals typically appeared within 5–10 d. Crystals were cryoprotected in the reservoir solution supplemented with 35% ethylene glycol and flash-frozen in liquid nitrogen.

### Crystallographic data collection and structure determination

Diffraction data were collected at the PXIII beamline Swiss Light Source, at −173 °C with a wavelength of 1.2783 Å. The crystal had a rod-like shape. With the goal of obtaining a strong anomalous signal arising from bound zinc atoms in the RING1, IBR and Rcat domains, 360° of data were collected at three different translations across the length of the crystal. The three independent datasets from the single crystal (1,080° of data total) were merged into a single reflection file using XSCALE. Data were indexed, integrated and scaled using XDS^51^. The structure was determined by single anomalous diffraction from zinc atoms, which were located using SHELXC/D/E^52^ followed by phase extension using PHENIX Autosol. The resultant map was used in Buccaneer^53^ for automatic chain tracing to build the initial model. Further iterative rounds of manual building and refinement were done using COOT^54^ and PHENIX^55^. Initially, waters were placed manually in unmodeled density observed in both the 2Fo–Fc map at 1σ and the Fo–Fc map at 3σ contour levels. Next, the ‘Update waters’ option in PHENIX was used to monitor, add and/or remove waters during the refinement. All waters in the final coordinate file were manually inspected to confirm density in the 2Fo–Fc map. In the final structure, the N-terminal residues of the construct (ARIH2 residues 51–57) and a loop connecting residues 128 and 138 were not visible in the density and hence were not modeled. Pro267 is modeled with a *cis* peptide bond in both molecules in the asymmetric unit. For the final model, the Clashscore is 6.2 and the Ramachandran statistics are 96% favored, 4% allowed and no outliers. Data collection and refinement statistics are listed in Supplementary Table 1.

### Cryo-EM sample preparation, imaging, data analysis and structure determination

#### Sample preparation

Cryo-EM samples were generated by mixing 10 µM neddylated CUL5-RBX2, 12 µM Vif–CBFβ-ELOBC, 14 µM A3C or A3G and 10 µM ARIH2* (L381A E382A E455A). The mixture was incubated at 4 °C for at least 30 min, and subjected to size-exclusion chromatography using a Superose 6 Increase column, in 25 mM HEPES, 100 mM NaCl, 1 mM DTT. Next, 3–4 μl of freshly assembled protein complex at 0.5 mg ml^−1^ was applied to glow discharged (30 s at medium intensity) Quantifoil holey carbon grids (R1.2/1.3 200 mesh) at 4 °C and 100% humidity. Grids were immediately blotted with Whatman no. 1 filter paper (blot time 3 s, blot force 4) and vitrified by plunging into liquid ethane using Vitrobot Mark IV (Thermo Fisher Scientific).

#### Data collection

Cryo-EM datasets were collected using SerialEM v.3.8.0-b5 (ref. ^56^) on a Titan Krios electron microscope at 300 kV with a Quantum-LS energy filter, using a K3 direct detector in counting mode. In total, 9,271 images were collected for the A3C complex and 7,830 images for the A3G complex at a pixel size of 0.8512 and 1.094 Å, respectively. The total exposure ranged from 75 to 90 e^−^ Å^−2^ and defocus values from −0.7 to −2.5 μm. The data collection statistics are listed in Supplementary Table 2.

#### Data processing

The micrographs were imported into RELION 3.1 (ref. ^57^), motion corrected and dose weighted using RELION’s own implementation of MotionCorr2 (ref. ^58^), followed by contrast transfer function estimation with GCTF v.1.06 (ref. ^59^).

For the reconstruction of A3C-bound neddylated CRL5^Vif-CBFβ^-ARIH2*, 5,030,529 particles were initially picked using Gautomatch v.056 (K. Zhang, MRC Laboratory of Molecular Biology), followed by two-dimensional (2D) and 3D classification. Initially, particles were binned by a factor of 5, resulting in 4.26 Å per pixel. Subsequent 2D and 3D classifications were done to remove particles belonging to poorly resolved classes. Cryo-EM data for both the A3C- and the A3G-bound neddylated CRL5^Vif-CBFβ^-ARIH2* assemblies refined to several similar but nonidentical classes. Even during initial processing, it was apparent that density corresponding to Vif-CBFβ-A3C was relatively poorly resolved, presumably due to heterogeneous orientations of these subunits relative to CUL5. One class with 7,689 particles showed features for the entire complex during 3D classification. This class refined to 6.8-Å resolution, and was low-pass filtered to 7.5 Å to enable docking of subunits (Extended Data Fig. 3a).

Two masks were used for initial consensus refinement, both encompassing the visible density for the entire CRL5^Vif-CBFβ^-A3C-ARIH2* complex (Extended Data Fig. 3a). The narrower mask showed more density for the Vif-CBFβ-A3C subunits, resulting in a reconstruction with global resolution of 3.7 Å. The portion of the map corresponding to neddylated CUL5-RBX2-ARIH2* showed clear density with distinct features. Focused 3D classification using a mask covering only ARIH2* bound to neddylated CUL5 (C-terminal region)-RBX2 was used to further improve reconstruction. After iterative rounds of classification, a final set of 191,792 particles were used for final refinement with full pixel size, resulting in a 3.4-Å reconstruction with well-resolved interfaces between ARIH2* and neddylated CUL5-RBX2. The flow chart shown in Extended Data Fig. 3a shows the data processing schemes, including for maps with density for all proteins in the A3C-bound neddylated CRL5^Vif-CBFβ^-ARIH2* at 6.8-Å resolution (shown low-pass filtered to 7.5-Å resolution in Fig. 1d and Extended Data Fig. 5a), and for the ARIH2* assembly with a portion of neddylated CUL5 at RBX2 at 3.4-Å resolution. Reported resolution is based on the gold-standard Fourier shell correlation using the 0.143 criterion (Extended Data Fig. 3b,c). Final maps were sharpened using RELION^57^ postprocessing or DeepEMhancer^40^. To facilitate model building by improved map quality by local sharpening and noise reduction, two half maps from the final refinement were provided without a mask as input to DeepEMhancer^40^. This resulted in reduced anisotropy and better overall map connectivity. The flow chart for A3G-bound complex processing is shown in Extended Data Fig. 4.

#### Model building and refinement

A number of maps were used as a guide for model building and refinement. Initially, coordinates from existing crystal structures were fit into the 6.8-Å resolution map (low-pass filtered to 7.5-Å resolution, Electron Microscopy Data Bank (EMDB) EMD-12998), which showed density for all subunits as follows: A3C (PDB ID 3VOW, ref. ^39^); Vif-CBFβ-ELOBC and CUL5 N-terminal domain (chains l, m, n, o and p from PDB ID 4N9F, ref. ^36^), the CR3 domain of CUL5 (PDB ID 6V9I), the crystal structure of ARIH2 from this study (PDB ID 7OD1), and domains from neddylated CUL5 C-terminal domain RBX1 (PDB ID 3DQV) split into two units (the 4HB and C/R domain were fit together as one unit, NEDD8 and its linked CUL5 H29-helix and WHB domain were fit together as another unit)^12^.

The coordinates for a neddylated CUL5-RBX2-ARIH2* subcomplex of the A3C-bound neddylated CRL5^Vif-CBFβ^-ARIH2* E3-E3 were subjected to rebuilding, guided by the map processed with DeepEMhancer, and refined using the 3.4-Å resolution postprocessed map arising from focused refinement (A3C E3-E3 catalytic focused in Extended Data Fig. 3a, EMD-12995)^40^. Initial regions of crystal structures were docked in the focus refined map using Chimera v.1.14 (ref. ^60^) and they were allowed to move independently of each other in rigid body refinements using PHENIX^55^. ARIH2*‘s UBAL, RING1 and RTI helix region was visible at relatively lower resolution in all cryo-EM maps. Thus, this region of the model was only further subjected to rigid body refinement. The remainder of the structure was subjected to manual model building (including converting the original RBX1 model into the RBX2 protein in this complex) using COOT^54^. Real space refinements were performed in an iterative manner to improve the fit using Phenix.refine^55^. Other than ARIH2* Rcat domain (residues 283–351), most parts of the complex could be resolved except residues 1–34, 51–53, 128–133 and 492–493 of ARIH2*, 6–27 of RBX2 and 1–151, 170–173, 189–193, 386–400 and 675–679 of CUL5, which are not modeled in the final structure. Because the ARIH2* UBAL, RING1 and RTI helix were less well-resolved in the maps, side chains were maintained largely during refinement by restraining them using reference model restraints based on the crystal structures of autoinhibited ARIH2 reported in this study. The side chain of CUL5 Glu717, which is not visible in the density but faces the center of the interface with NEDD8, was modeled based on the crystal structure showing the corresponding portion of neddylated CUL5 (PDB ID 3DQV)^12^, but with zero occupancy. Structures and maps in the figures were rendered with PyMOL or ChimeraX v.1.0.

### Analysis of published HDX–mass spectrometry data based on structures of autoinhibited ARIH2 and the ARIH2* complex with neddylated CUL5-RBX2

A previous study compared HDX, quantified by mass spectrometry of tryptic peptides generated after HDX was quenched, for CUL5-RBX2-ELOBC, NEDD8–CUL5-RBX2-ELOBC, ARIH2 and ARIH2-NEDD8–CUL5-RBX2-ELOBC^33^. The authors compared HDX properties between CUL5-RBX2-ELOBC and NEDD8–CUL5-RBX2-ELOBC, between ARIH2 and ARIH2-NEDD8–CUL5-RBX2-ELOBC, and between NEDD8–CUL5-RBX2-ELOBC and ARIH2-NEDD8–CUL5-RBX2-ELOBC and determined peptides that after 0.25 min of HDX showed either statistically significant protection or deprotection on complex formation^33^. Because HDX–mass spectrometry data serve as an independent test of changes in protein conformation, the peptide sequences that show statistically significant protection or deprotection on complex formation were color-coded on the structures of the individual CUL5-RBX2 (ref. ^22^) and autoinhibited ARIH2 E3s, and the neddylated CRL5^Vif-CBFβ^-ARIH2* E3-E3 complex (deprotected red, protected-blue, no significant difference white and sequences not detected in the experiments black, Supplementary Video 1). Morphs showing potential trajectories between different conformations were generated using Chimera^60^, and movies were made using PyMOL (The PyMOL Molecular Graphics System, v.2.0 Schrödinger, LLC).

### Reporting Summary

Further information on research design is available in the Nature Research Reporting Summary linked to this article.

## Online content

Any methods, additional references, Nature Research reporting summaries, source data, extended data, supplementary information, acknowledgements, peer review information; details of author contributions and competing interests; and statements of data and code availability are available at 10.1038/s41589-021-00858-8.

## Supplementary information

Supplementary Information Supplementary Tables 1 and 2.

Reporting Summary

Supplementary Video Morphs showing conformational differences between unneddylated CUL5-RBX2, autoinhibited ARIH2 and the neddylated CRL5-ARIH2 E3-E3 assembly.

## Data Availability

Cryo-EM maps and structural coordinates will be available from the EMDB and RCSB on publication. The EM maps and corresponding models were deposited in the RCSB and EMDB with accession codes PDB IDs 7OD1 and 7ONI, and EMD-12995 (with DeepEMhancer map as additional map with this accession code), EMD-12998, EMD-12999 (with DeepEMhancer map as additional map with this accession code), EMD-13000 and EMD-13001. Publicly available datasets used in this study are: PDB IDs 3VOW, 4N9F, 6V9I, 3DQV, 3DPL, 7B5L, 5EDV, 5CAW, 5N2W and 4B9M. Source data are provided with this paper.

## Source data

Source Data Fig. 2 Unprocessed gels.
Source Data Fig. 3 Unprocessed gels.
Source Data Fig. 4 Unprocessed gels.
Source Data Fig. 5 Unprocessed gels.
Source Data Extended Data Fig. 1 Unprocessed gels.
Source Data Extended Data Fig. 2 Unprocessed gels.
Source Data Extended Data Fig. 7 Unprocessed gels.
Source Data Extended Data Fig. 8 Unprocessed gels.

## Extended data

are available for this paper at 10.1038/s41589-021-00858-8.
