# Supplementary material for: CUL5-ARIH2 E3-E3 ubiquitin ligase structure reveals cullin-specific NEDD8 activation
Source: Nat Chem Biol. 2021 Sep 13;17(10):1075–83. doi: 10.1038/s41589-021-00858-8 (PMC8460447; doi:10.1038/s41589-021-00858-8)
Supplement: Supplementary file 1 — Supplementary Tables 1 and 2. [file 41589_2021_858_MOESM1_ESM.pdf]

nature portfolio

https://doi.org/10.1038/s41589-021-00858-8

Supplementary information

CUL5-ARIH2 E3-E3 ubiquitin ligase structure reveals cullin-specific NEDD8 activation

In the format provided by the authors and unedited

Supplementary Table 1. Crystallographic data collection and refinement statistics

	Autoinhibited ARIH2
**Data collection**	
Space group	P 1 21 1
Cell dimensions	
a, b, c (Å)	77.57 80.54 92.09
α, β, γ (°)	90 105.76 90
Resolution (Å)	66.7 - 2.45 (2.54 - 2.45)*
R_{sym} or R_{merge}	0.13 (2.4)
I / σI	19.5 (1.0)
Completeness (%)	95.9 (75.0)
Redundancy	18.6 (11.2)
**Refinement**	
Resolution (Å)	2.45
No. reflections	38685 (2975)
R_{work} / R_{free}	22.1 / 26.1
No. atoms	6972
Protein	6940
Ligand/ion	12
Water	20
B-factors	
Protein	76.9
Ligand/ion	81.4
Water	60.9
R.m.s. deviations	
Bond lengths (Å)	0.003
Bond angles (°)	0.56

*Values in parentheses are for highest-resolution shell.

Supplemental Table 2. Cryo-EM data collection, refinement and validation statistics

	Neddylated CRL5$^{Vif\text{-}CBF\beta}$-ARIH2*-APOBEC3C	Neddylated CRL5$^{Vif\text{-}CBF\beta}$-ARIH2*-APOBEC3C	Neddylated CUL5 C-terminal region-RBX2-ARIH2*	Neddylated CRL5$^{Vif\text{-}CBF\beta}$-ARIH2*-APOBEC3G	Neddylated CUL5 C-terminal region-RBX2-ARIH2*
Map name in processing pipeline	A3C consensus	A3C full complex consensus	A3C E3-E3 catalytic focused	A3G consensus	A3G E3-E3 catalytic focused
Accession codes	EMD-13000	EMD-12998	EMD-12995 PDB 7ONI	EMD-13001	EMD-12999
**Data collection and processing**					
Microscope/detector	Krios/K3	Krios/K3	Krios/K3	Krios/K3	Krios/K3
Magnification	105,000	105,000	105,000	81,000	81,000
Voltage (kV)	300	300	300	300	300
Electron exposure (e–/Å^2)	14.9	14.9	14.9	15	15
Defocus range (μm)	0.7 ~ 2.5	0.7 ~ 2.5	0.7 ~ 2.5	0.7 ~ 2.5	0.7 ~ 2.5
Pixel size (Å)	0.8512	0.8512	0.8512	1.094	1.094
Symmetry imposed	n/a	n/a	n/a	n/a	n/a
Initial particle images (no.)	5,030,529	5,030,529	5,030,529	1,200,719	1,200,719
Final particle images (no.)	191,538	7,689	191,792	194,585	194,585
Map resolution (Å)	3.7	6.8	3.4	3.8	3.5
FSC threshold					
Map resolution range (Å)	3.3 – 8.2	-	3.2 – 6.2	3.4 – 8.0	3.3 – 6.3
**Refinement**					
Initial model used (PDB code)			3DQV 7OD1		
Model resolution (Å)			3.40		
FSC threshold			0.143		
Map sharpening *B* factor (Å^2)			-70		
Model composition					
Non-hydrogen atoms			8840		
Protein residues			1141		
Ligands			7		
B factors (Å^2)					
Protein			99		
Ligand			155		
R.m.s. deviations					
Bond lengths (Å)			0.003		
Bond angles (°)			0.529		
Validation					
MolProbity score			1.7		
Clashscore			7.1		
Poor rotamers (%)			0		
Ramachandran plot					
Favored (%)			96		
Allowed (%)			4		
Disallowed (%)			0		
