# Supplementary material for: CUL5-ARIH2 E3-E3 ubiquitin ligase structure reveals cullin-specific NEDD8 activation
Source: Nat Chem Biol. 2021 Sep 13;17(10):1075–83. doi: 10.1038/s41589-021-00858-8 (PMC8460447; doi:10.1038/s41589-021-00858-8)
Supplement: Supplementary file 2 — Reporting Summary [file 41589_2021_858_MOESM2_ESM.pdf]

nature research

Corresponding author(s): Brenda Schulman

Last updated by author(s): Jun 24, 2021

Reporting Summary

Nature Research wishes to improve the reproducibility of the work that we publish. This form provides structure for consistency and transparency in reporting. For further information on Nature Research policies, see Authors & Referees and the Editorial Policy Checklist.

Statistics

For all statistical analyses, confirm that the following items are present in the figure legend, table legend, main text, or Methods section.

n/a	Confirmed	
☐	☒	The exact sample size (*n*) for each experimental group/condition, given as a discrete number and unit of measurement
☐	☒	A statement on whether measurements were taken from distinct samples or whether the same sample was measured repeatedly
☒	☐	The statistical test(s) used AND whether they are one- or two-sided<br>*Only common tests should be described solely by name; describe more complex techniques in the Methods section.*
☒	☐	A description of all covariates tested
☒	☐	A description of any assumptions or corrections, such as tests of normality and adjustment for multiple comparisons
☒	☐	A full description of the statistical parameters including central tendency (e.g. means) or other basic estimates (e.g. regression coefficient) AND variation (e.g. standard deviation) or associated estimates of uncertainty (e.g. confidence intervals)
☒	☐	For null hypothesis testing, the test statistic (e.g. *F*, *t*, *r*) with confidence intervals, effect sizes, degrees of freedom and *P* value noted<br>*Give P values as exact values whenever suitable.*
☒	☐	For Bayesian analysis, information on the choice of priors and Markov chain Monte Carlo settings
☒	☐	For hierarchical and complex designs, identification of the appropriate level for tests and full reporting of outcomes
☒	☐	Estimates of effect sizes (e.g. Cohen's *d*, Pearson's *r*), indicating how they were calculated

Our web collection on statistics for biologists contains articles on many of the points above.

Software and code

Policy information about availability of computer code

Data collection	Gel imaging: Amersham Imager 600, Amersham Typhoon; Cryo-EM: SerialEM v3.8.0-b5, FEI EPU v2.7.0
Data analysis	Assay analysis: Graph Pad Prism v8.4.1 and v8.3 , Microsoft Excel v16.16.25, ImageQuant TL v8.2.0.0; Cryo-EM: RELION 3.1, Gautomatch v.056, Gctf v1.06, MotionCorr2 v1.2.6; Structure visualization: Chimera v1.14, ChimeraX 1.0, PyMol 1.5.0.4; Model Building: COOT 0.8.9.2, Phenix.refine 1.18.2, SHELXC/D/E, DECA (github.com/komiveslab/DECA)

For manuscripts utilizing custom algorithms or software that are central to the research but not yet described in published literature, software must be made available to editors/reviewers. We strongly encourage code deposition in a community repository (e.g. GitHub). See the Nature Research guidelines for submitting code & software for further information.

Data

Policy information about availability of data

All manuscripts must include a data availability statement. This statement should provide the following information, where applicable:

- Accession codes, unique identifiers, or web links for publicly available datasets
- A list of figures that have associated raw data
- A description of any restrictions on data availability

Raw gels are provided as supplementary information (Source Data Fig. 1-4 and Source Data Extended Data Fig. 1-4). Figures with associated raw data: Figure 2,3,4 and 5 and Extended Data Figures 1, 2, 7 and 8
The EM maps and corresponding models were deposited in the RCSB and EMDB with accession codes PDB ID: 7OD1 and 7ONI, and EMD-12995 (with DeepEMhancer map as additional map with this accession code), EMD-12998, EMD-12999 (with DeepEMhancer map as additional map with this accession code), EMD-13000 and EMD-13001. Publicly available datasets used in this study: PDB ID: 3VOW, PDB ID: 4N9F, PDB ID: 6V9I, PDB ID: 3DQV, PDB ID: 3DPL, PDB ID: 7B5L, PDB ID: 5EDV, PDB ID: 5CAW, PDB ID: 5N2W, PDB ID: 4B9M

Field-specific reporting

Please select the one below that is the best fit for your research. If you are not sure, read the appropriate sections before making your selection.

☒ Life sciences ☐ Behavioural & social sciences ☐ Ecological, evolutionary & environmental sciences

For a reference copy of the document with all sections, see nature.com/documents/nr-reporting-summary-flat.pdf

Life sciences study design

All studies must disclose on these points even when the disclosure is negative.

Sample size	Sample size calculations were not performed. Based on previous experience, at least two independent replicates were carried out for all functional assays.
Data exclusions	Cryo-EM data was processed using Relion, which excluded low-quality data to reach high-resolution using statistical methods. 2D and 3D classification were used for particle selection. The exclusion criteria is pre-established as implementation in Relion, a common practice in cryo-EM.
Replication	All experiments were performed at least twice and independent from eachother. All attempts at replication were successful and the results were reproducible.
Randomization	Randomization is not required based on the nature of structural biology.
Blinding	Blinding was not performed based on the nature of strucutal biology.

Reporting for specific materials, systems and methods

We require information from authors about some types of materials, experimental systems and methods used in many studies. Here, indicate whether each material, system or method listed is relevant to your study. If you are not sure if a list item applies to your research, read the appropriate section before selecting a response.

Materials & experimental systems

n/a	Involved in the study	
☒	☐	Antibodies
☐	☒	Eukaryotic cell lines
☒	☐	Palaeontology
☒	☐	Animals and other organisms
☒	☐	Human research participants
☒	☐	Clinical data

Methods

n/a	Involved in the study	
☒	☐	ChIP-seq
☒	☐	Flow cytometry
☒	☐	MRI-based neuroimaging

Eukaryotic cell lines

Policy information about cell lines

Cell line source(s)	High five cell (BTI-TN-5B1-4) were obtained from ThermoFisher Scentific (catalogue number:B85502).
Authentication	Cell lines were not authenticated.
Mycoplasma contamination	Cell lines were not tested for mycoplasma contamination
Commonly misidentified lines (See ICLAC register)	No commonly misidentified cell lines were used in this study.
