# Supplementary figures and images for: CUL5-ARIH2 E3-E3 ubiquitin ligase structure reveals cullin-specific NEDD8 activation
Source: Nat Chem Biol. 2021 Sep 13;17(10):1075–83. doi: 10.1038/s41589-021-00858-8 (PMC8460447; doi:10.1038/s41589-021-00858-8)

Figure 2e

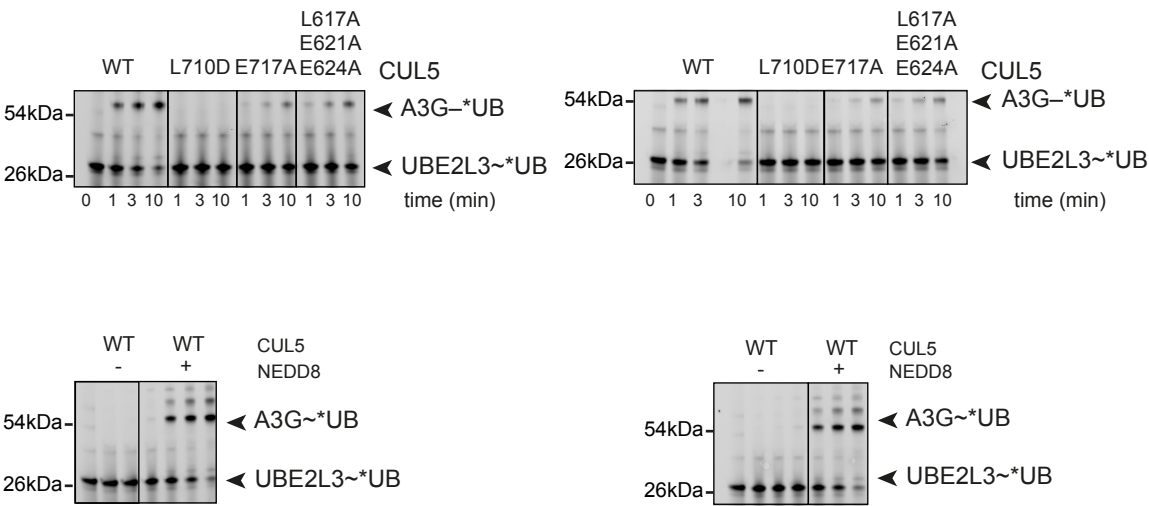

Figure 2e

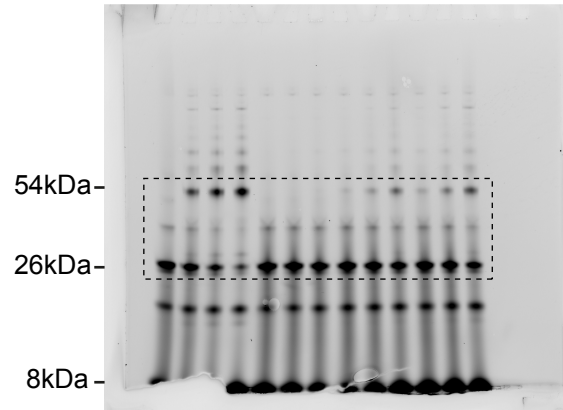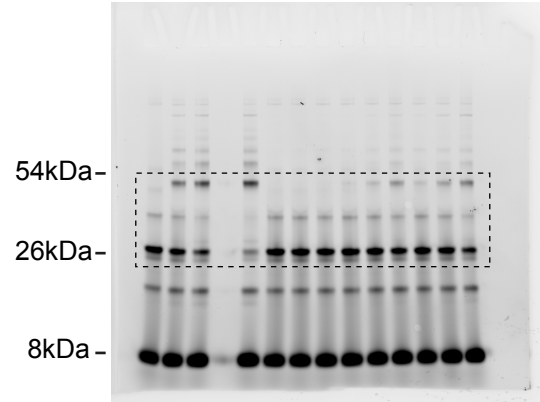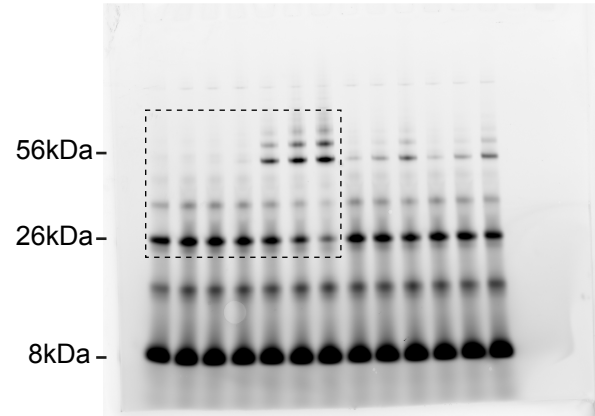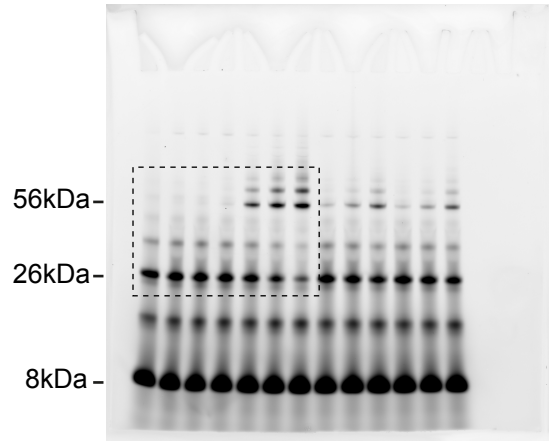

Supplement: Source Data Fig. 2 — Unprocessed gels. [file 41589_2021_858_MOESM4_ESM.pdf]

Figure 3g

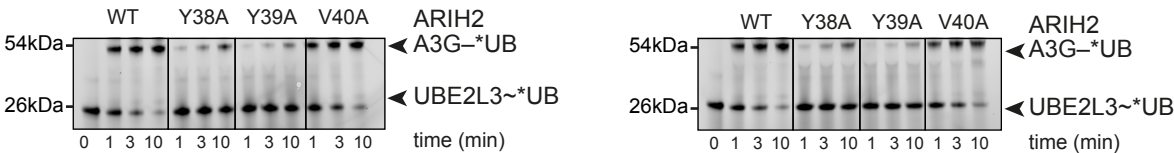

Figure 3h

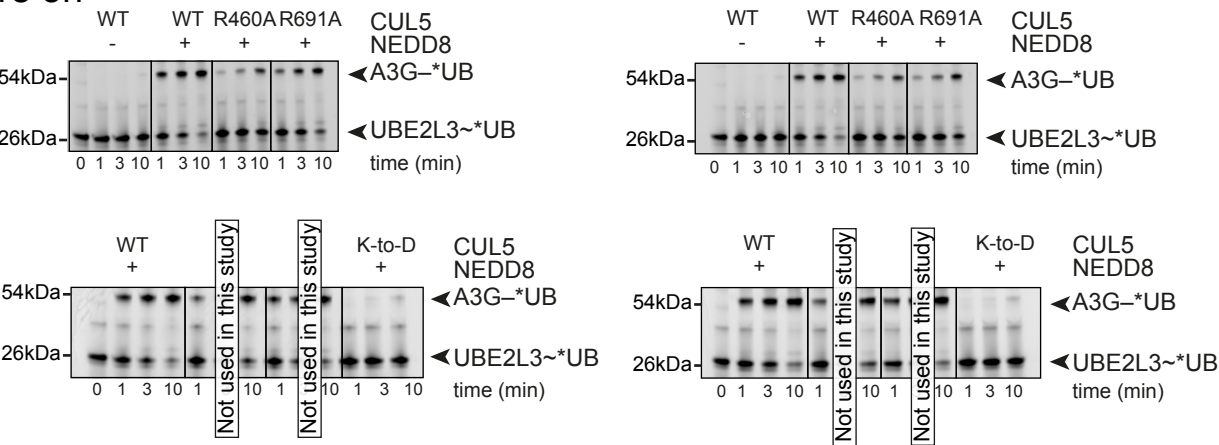

Figure 3g

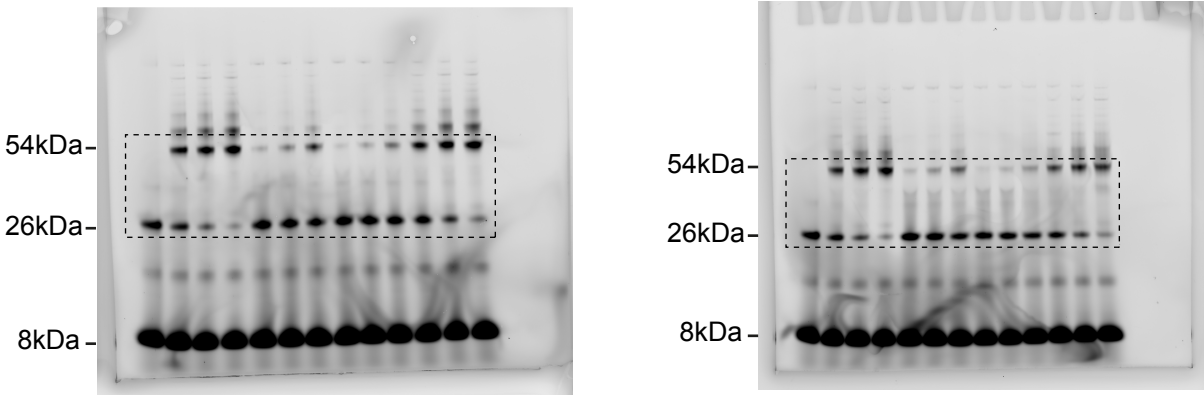

Figure 3h

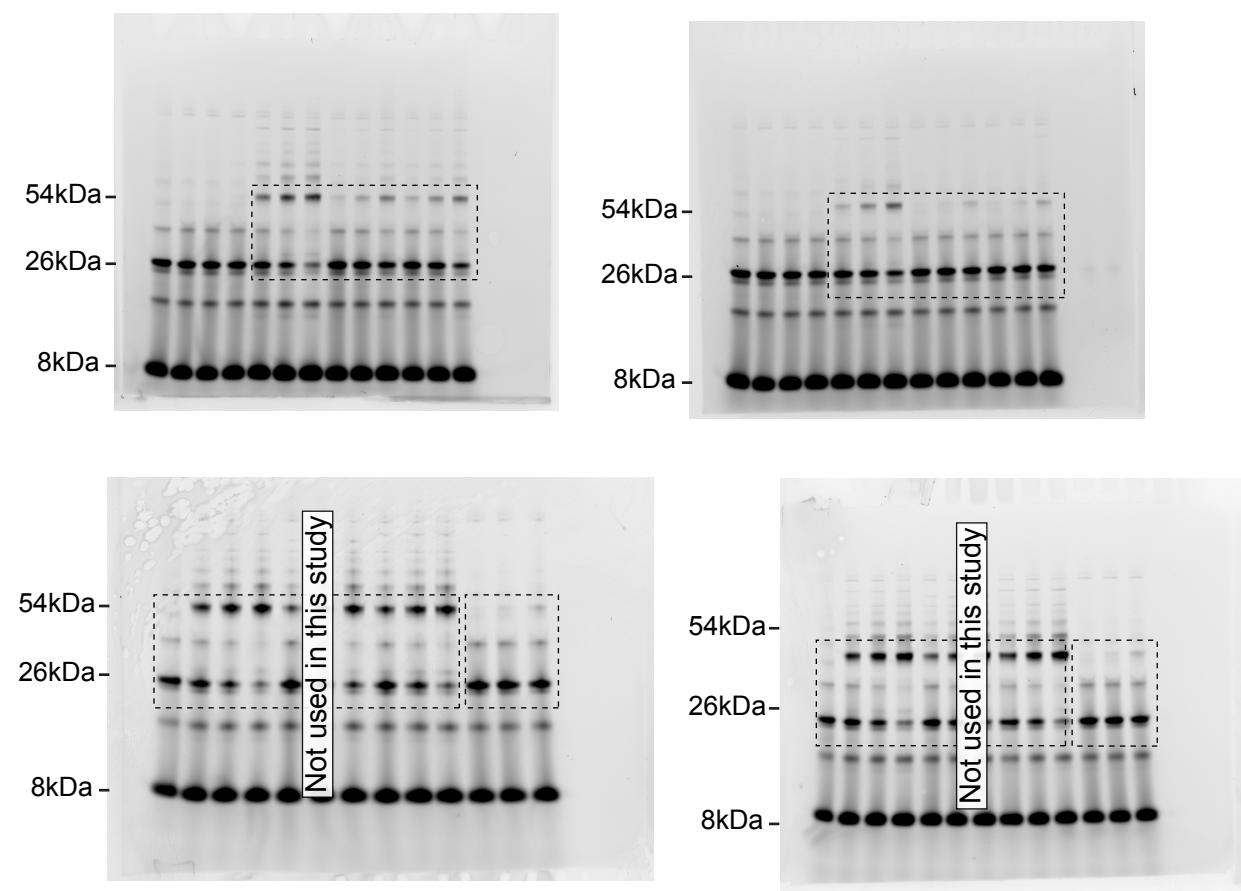

Supplement: Source Data Fig. 3 — Unprocessed gels. [file 41589_2021_858_MOESM5_ESM.pdf]

Figure 4e

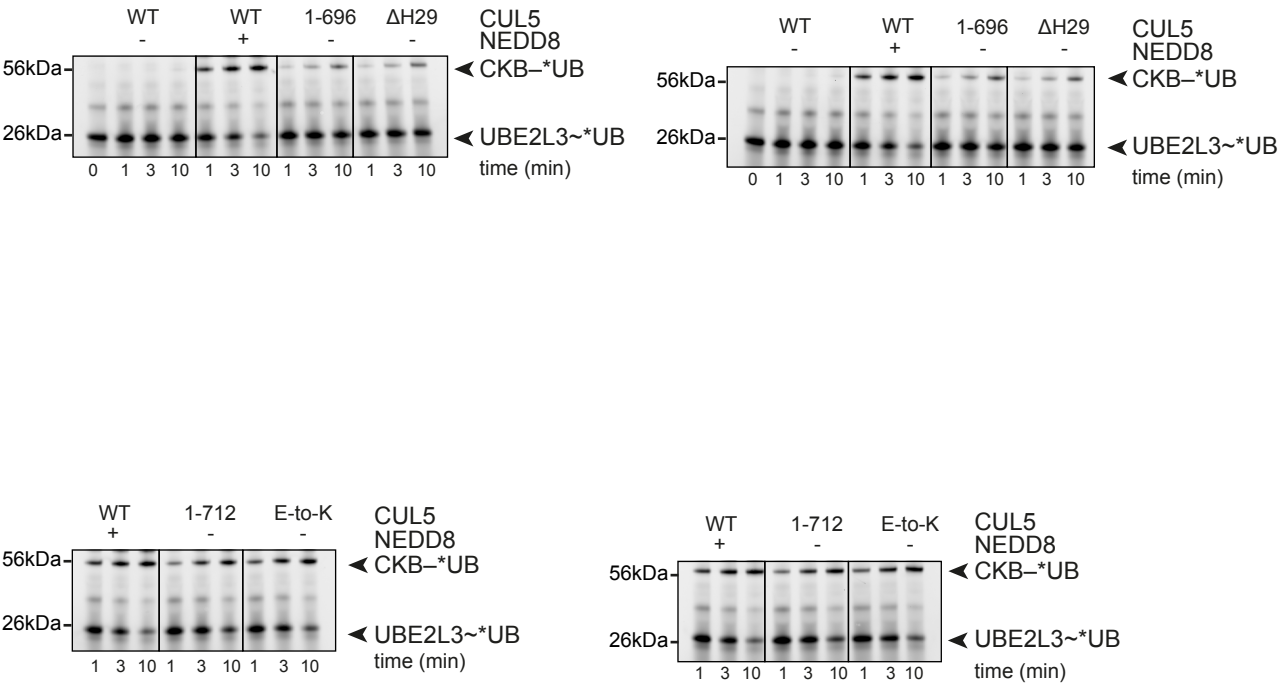

Figure 4e

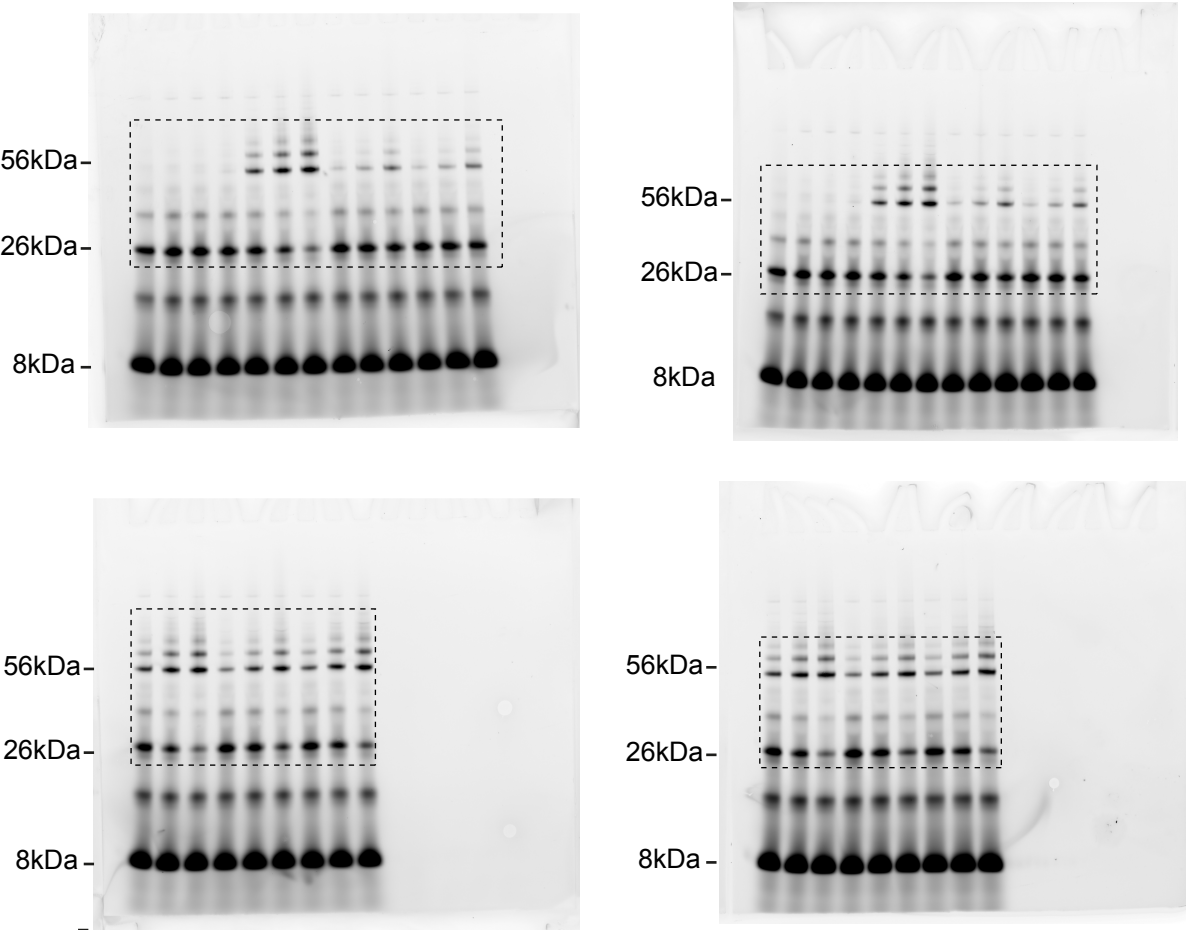

Supplement: Source Data Fig. 4 — Unprocessed gels. [file 41589_2021_858_MOESM6_ESM.pdf]

Figure 5e

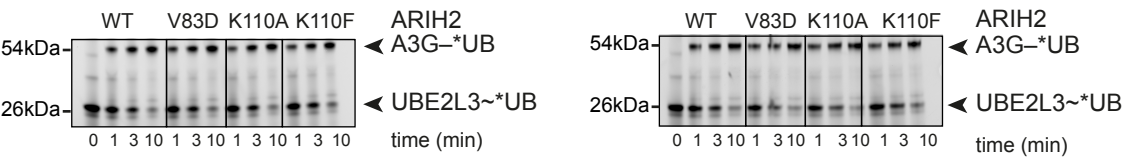

Figure 5e

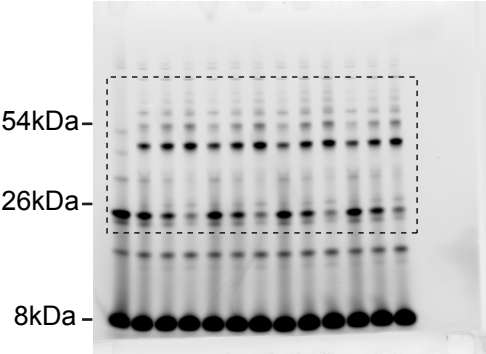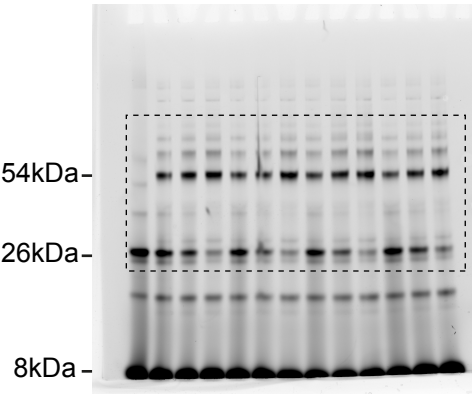

Supplement: Source Data Fig. 5 — Unprocessed gels. [file 41589_2021_858_MOESM7_ESM.pdf]

Extended Data Fig. 1c

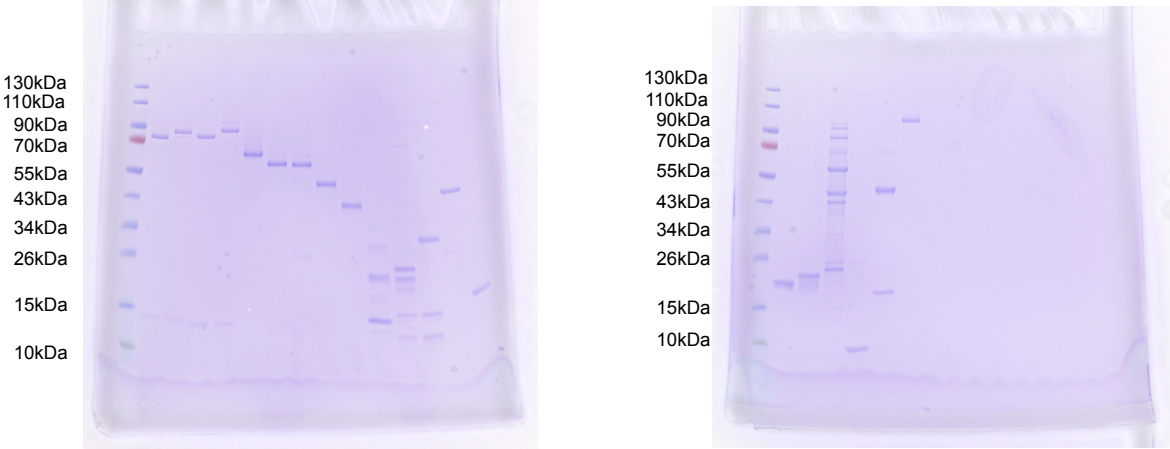

Extended Data Fig. 1e

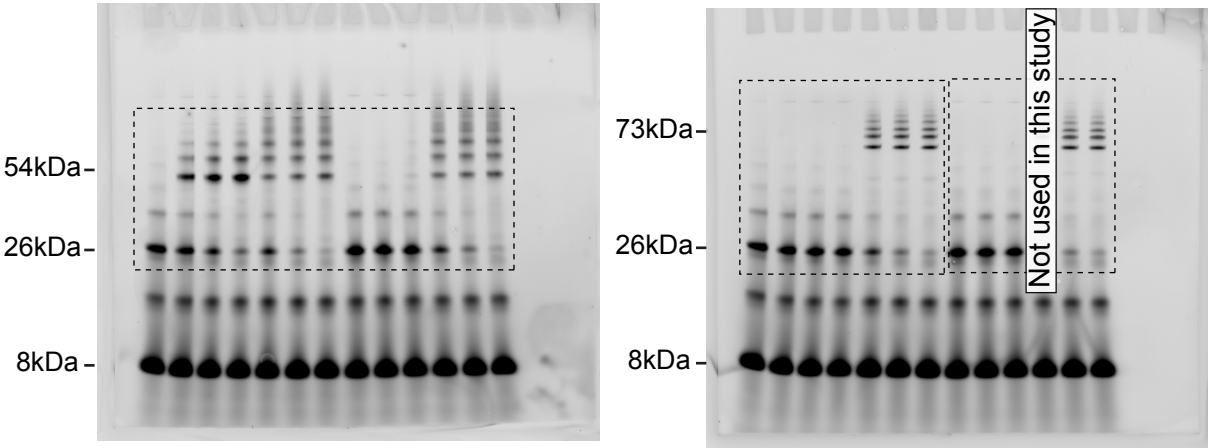

Extended Data Fig. 1f

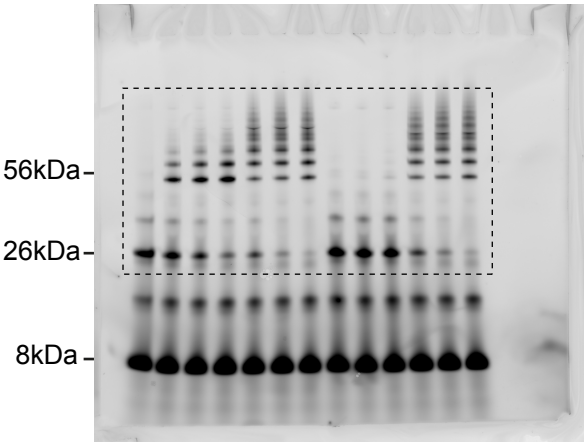

Extended Data Fig. 1g

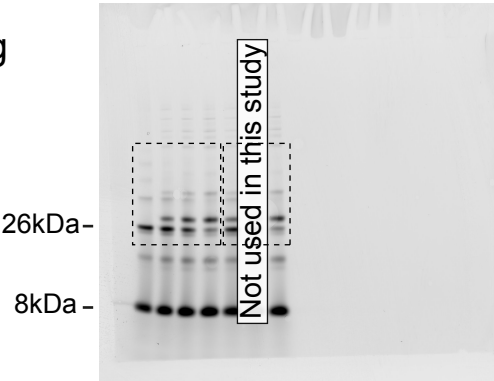

Supplement: Source Data Extended Data Fig. 1 — Unprocessed gels. [file 41589_2021_858_MOESM8_ESM.pdf]

Extended Data Fig. 2a

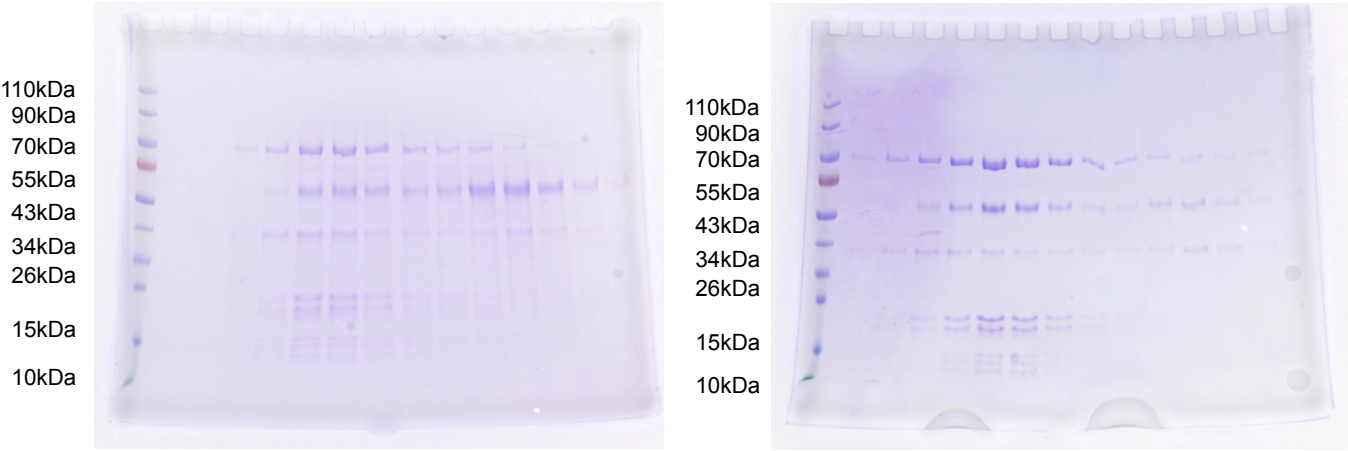

Extended Data Fig. 2f

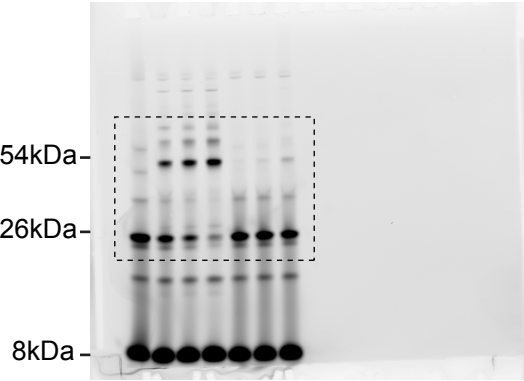

Supplement: Source Data Extended Data Fig. 2 — Unprocessed gels. [file 41589_2021_858_MOESM9_ESM.pdf]

Extended Data Fig 8a

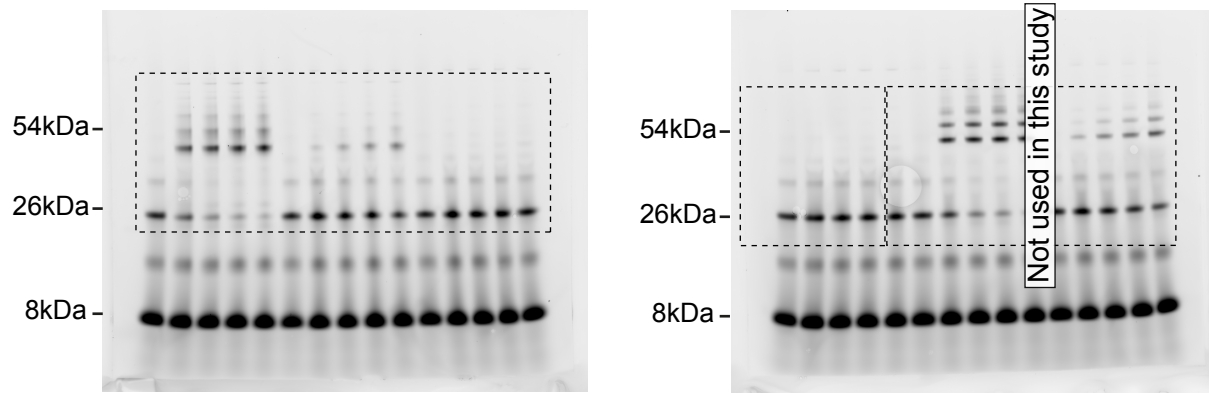

Extended Data Fig. 8b

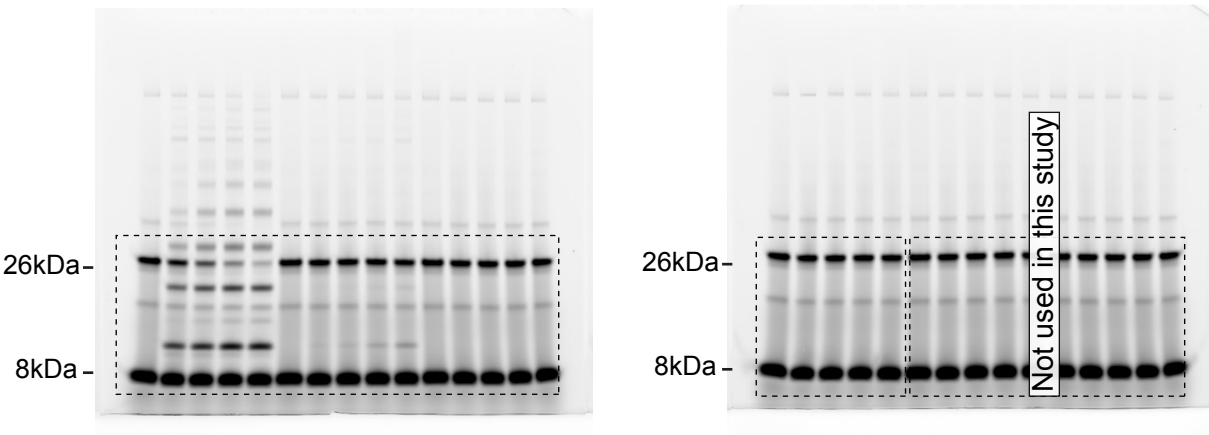

Supplement: Source Data Extended Data Fig. 8 — Unprocessed gels. [file 41589_2021_858_MOESM11_ESM.pdf]
