# Supplementary material for: CUL5-ARIH2 E3-E3 ubiquitin ligase structure reveals cullin-specific NEDD8 activation
Source: Nat Chem Biol. 2021 Sep 13;17(10):1075–83. doi: 10.1038/s41589-021-00858-8 (PMC8460447; doi:10.1038/s41589-021-00858-8)

Extended Data Figure 7a

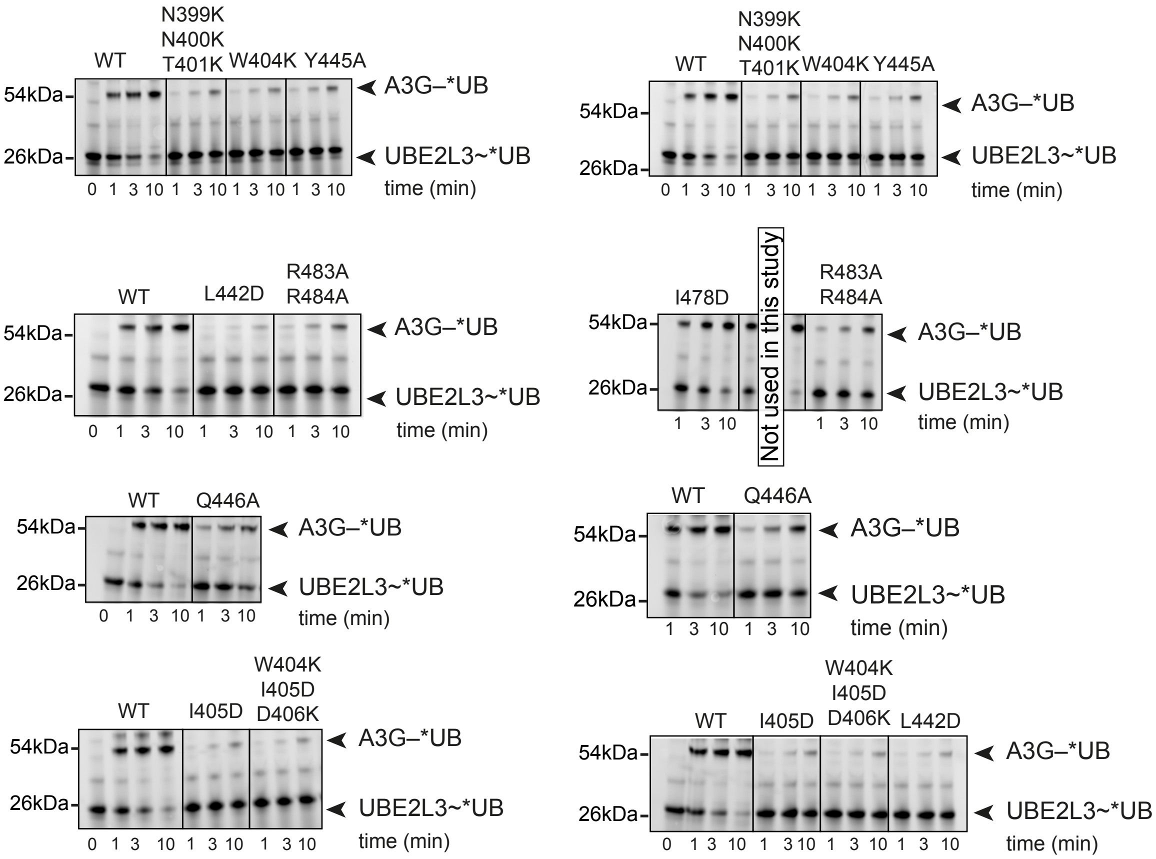

Extended Data Figure 7a

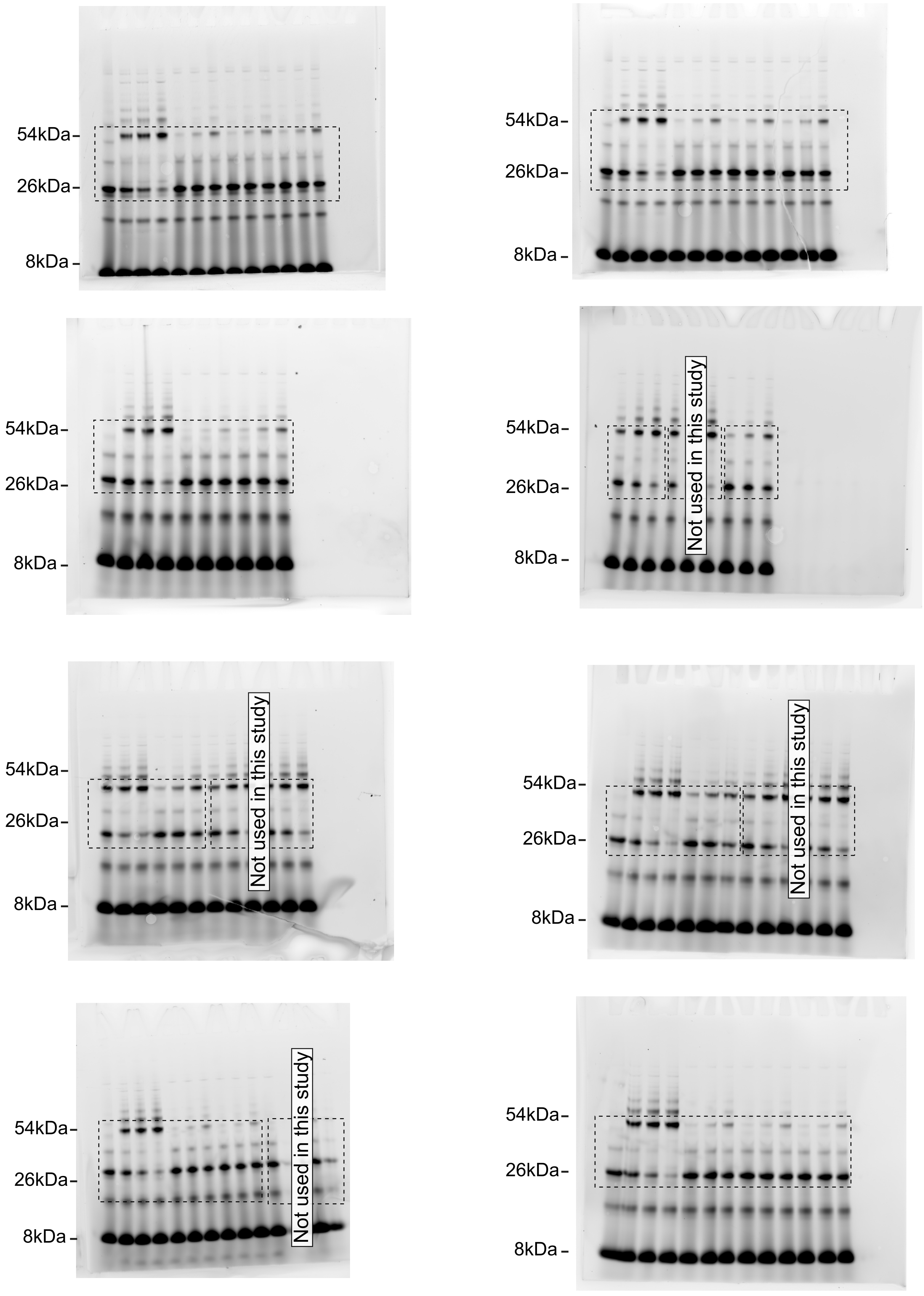

Extended Data Fig. 7d

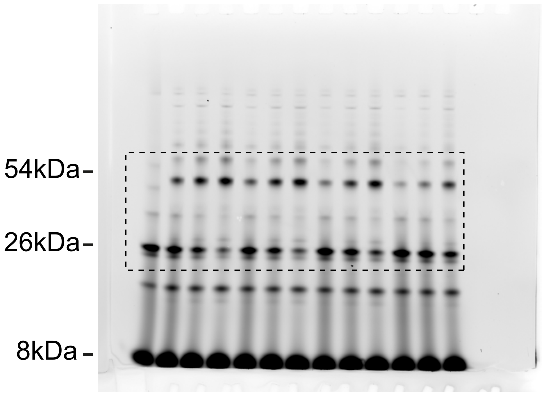

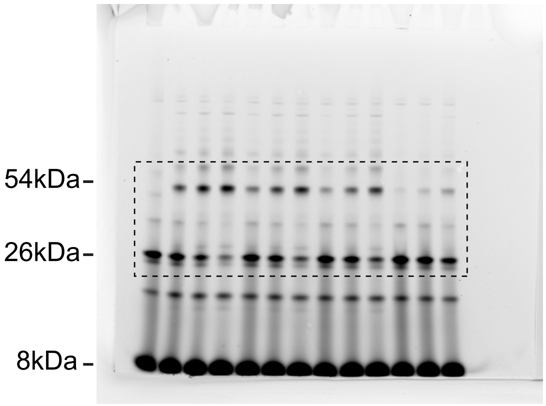

Extended Data Fig. 7e

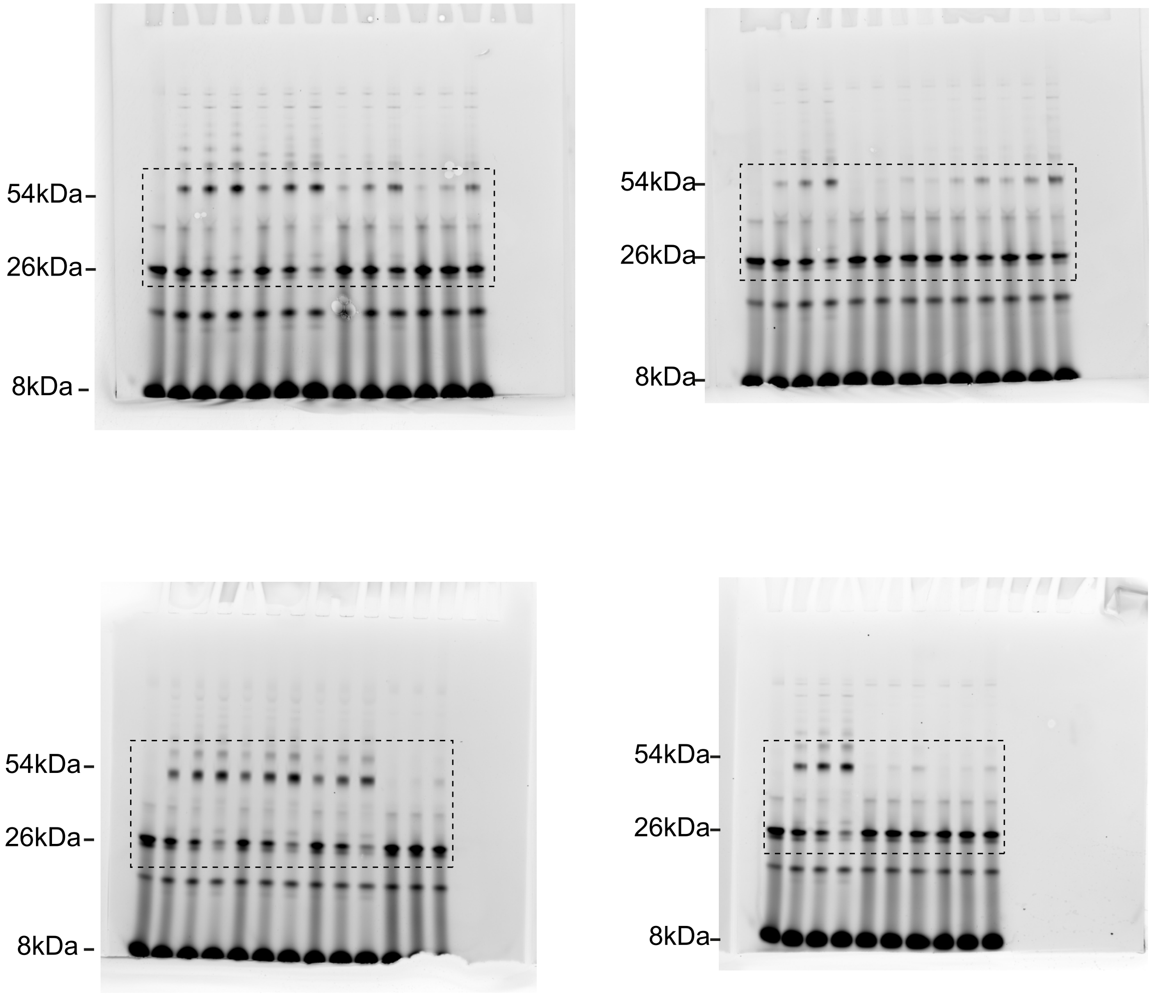

Extended Data Fig. 7f

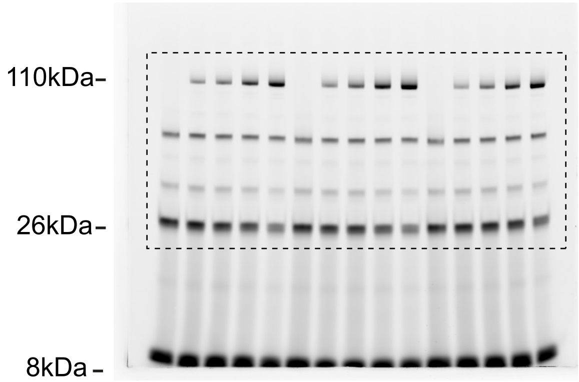

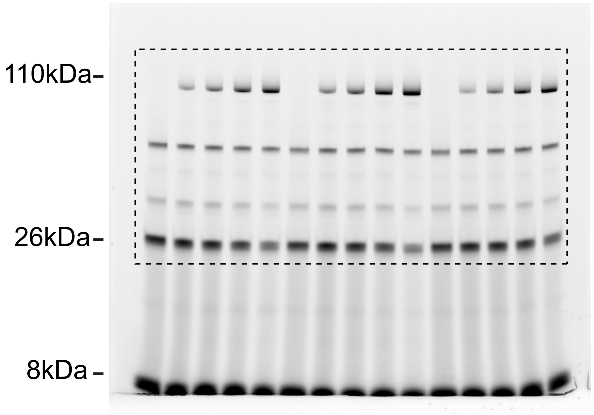

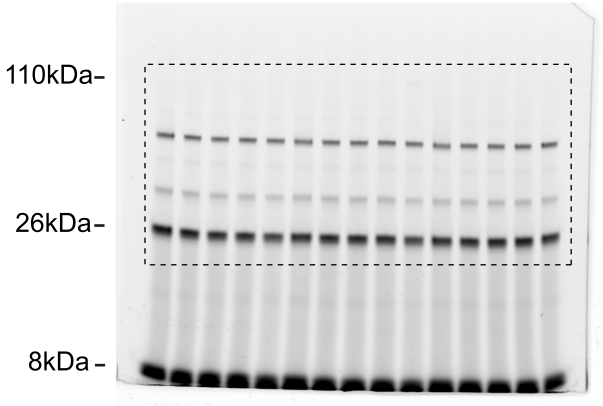

Supplement: Source Data Extended Data Fig. 7 — Unprocessed gels. [file 41589_2021_858_MOESM10_ESM.pdf]
